# Uncertainty‐Aware Deep Ensembles for Robust and Reliable Chemical Sensor Arrays

**DOI:** 10.1002/advs.76134

**Published:** 2026-06-15

**Authors:** Sungwoo Eo, Ji‐Hwan Eum, Suk‐Jeong Kwon, Minji Sagong, Dongchan Kim, Dong‐Ha Kim

**Affiliations:** ^1^ Department of Materials Science and Chemical Engineering Hanyang University Ansan Republic of Korea; ^2^ Department of Artificial Intelligence Hanyang University Ansan Republic of Korea

**Keywords:** deep ensemble, halitosis, multi‐sensor array, photothermal effect, uncertainty

## Abstract

Selective detection of sulfur‐containing gases such as hydrogen sulfide (H_2_S), methyl mercaptan (CH_3_SH), and dimethyl sulfide ((CH_3_)_2_S) is important for halitosis‐related breath and environmental monitoring. However, conventional metal‐oxide chemiresistive sensors show high cross‐reactivity toward these chemically similar species, which limits their practical selectivity. To address this, we present a deep‐ensemble‐assisted electronic nose based on catalytic metal nanoparticles‐decorated metal oxide nanofiber arrays. A 15‐channel multi‐sensor array was fabricated by anchoring diverse metal catalysts (Pt, Pd, Ir, and Co) onto metal oxide nanofiber scaffolds (SnO_2_, Co_3_O_4_, and WO_3_) through an intense pulsed light based photothermal process. Based on comprehensive datasets collected from a 15‐channel multi‐sensor array under varying gas species, concentrations, operating temperatures, and humidity levels, we developed a deep‐ensemble learning framework. This architecture simultaneously performs gas‐species classification and concentration quantification while providing predictive uncertainty, thereby demonstrating a proof‐of‐concept reliability‐aware platform for VSC sensing with potential relevance to future breath‐related and environmental monitoring applications.

## Graphical abstract

A reliability‐aware electronic nose is developed using photothermally anchored metal‐catalyst decorated metal oxide nanofiber sensor arrays combined with deep ensemble learning. Diverse catalytic nanofiber channels generate gas‐specific response patterns, enabling selective identification and quantification of sulfur‐containing gases. Predictive uncertainty estimation further ensures robust, trustworthy sensing under varying environmental conditions.

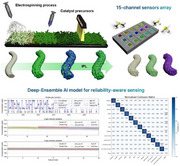

## 1 Introduction

Volatile sulfur compounds (VSCs), including hydrogen sulfide (H_2_S), methyl mercaptan (CH_3_SH), and dimethyl sulfide ((CH_3_)_2_S), are associated with halitosis and several metabolic and inflammatory disorders [1, 2, 3, 4, 5]. Accordingly, the development of reliable sensors capable of detecting these trace‐level species in exhaled breath has received considerable attention. Metal‐oxide semiconductor (MOS) gas sensors, such as SnO_2_, Co_3_O_4_, and WO_3_, have been widely explored for this purpose because of their high sensitivity, thermal stability, and compatibility with low‐cost fabrication [6, 7, 8, 9]. Indeed, MOS materials exhibit inherently strong reactivity toward sulfur‐containing gases, making them attractive candidates for VSCs detection. However, despite their high sensitivity, conventional MOS sensors exhibit poor selectivity toward structurally similar sulfur molecules. Because the three VSCs share broadly similar physicochemical characteristics and often undergo overlapping surface reactions on metal oxide sensors, their response patterns partially intersect, making selective discrimination challenging [10, 11].

To overcome the intrinsic selectivity limitations of single MOS sensors, recent studies have shifted toward a multi‐sensor array architecture coupled with data‐driven analysis techniques [10, 12, 13, 14]. By incorporating several sensing channels, each with distinct materials, nanostructures, or catalytic functionalization, e‐nose platforms attempt to expand the chemical–response space and capture subtle gas‐specific patterns [10, 12, 13]. Modern deep‐learning architectures, including convolutional, recurrent, and attention‐based neural networks, can capture rich spatiotemporal features from complex resistance dynamics, enabling substantially enhanced classification and regression performance [2, 12, 13, 15]. For instance, Cho et al. demonstrated micro‐LED modulated photoactivated sensors combined with deep CNNs for multi‐gas recognition [12], while Wang et al. introduced large‐scale biomimetic olfactory chips capable of recognizing complex odor mixtures using neural networks [13]. Despite the widespread adoption of AI, most deep learning‐based gas identification frameworks still generate deterministic predictions without quantifying uncertainty. However, achieving reliable VSC discrimination requires both uncertainty‐aware AI models and a sensing platform capable of generating sufficiently diverse and gas‐specific response signatures.

Because the three VSCs undergo highly similar adsorption‐oxidation pathways on conventional MOS surfaces, expanding the material heterogeneity through variations in catalyst composition and oxide chemistry is essential for providing the discriminative features needed for robust classification [10, 16]. In this study, we leverage ultrafast intense pulsed light (IPL)‐driven photothermal anchoring to develop a 15‐channel catalyst‐functionalized MOS nanofibers (NFs) sensor array, in which Pt, Pd, Ir, and Co nanoparticles (NPs) are rapidly integrated onto SnO_2_, Co_3_O_4_, and WO_3_ NFs [6, 16, 17]. Building on this sensor array platform, we further introduce a deep‐ensemble Transformer framework that performs robust gas‐species classification and concentration regression while explicitly estimating predictive uncertainty, thereby addressing the lack of reliability awareness in existing AI‐based gas‐identification systems. By integrating ultrafast materials diversification with reliability‐aware AI, this work presents a proof‐of‐concept platform for uncertainty‐aware VSC sensing and provides a basis for future breath‐related and environmental monitoring studies after further validation.

## 2 Results and Discussion

### 2.1 Catalytic Metal Nanoparticles Decorated Metal Oxide Nanofibers‐Based Sensors Array

**FIGURE 1:**
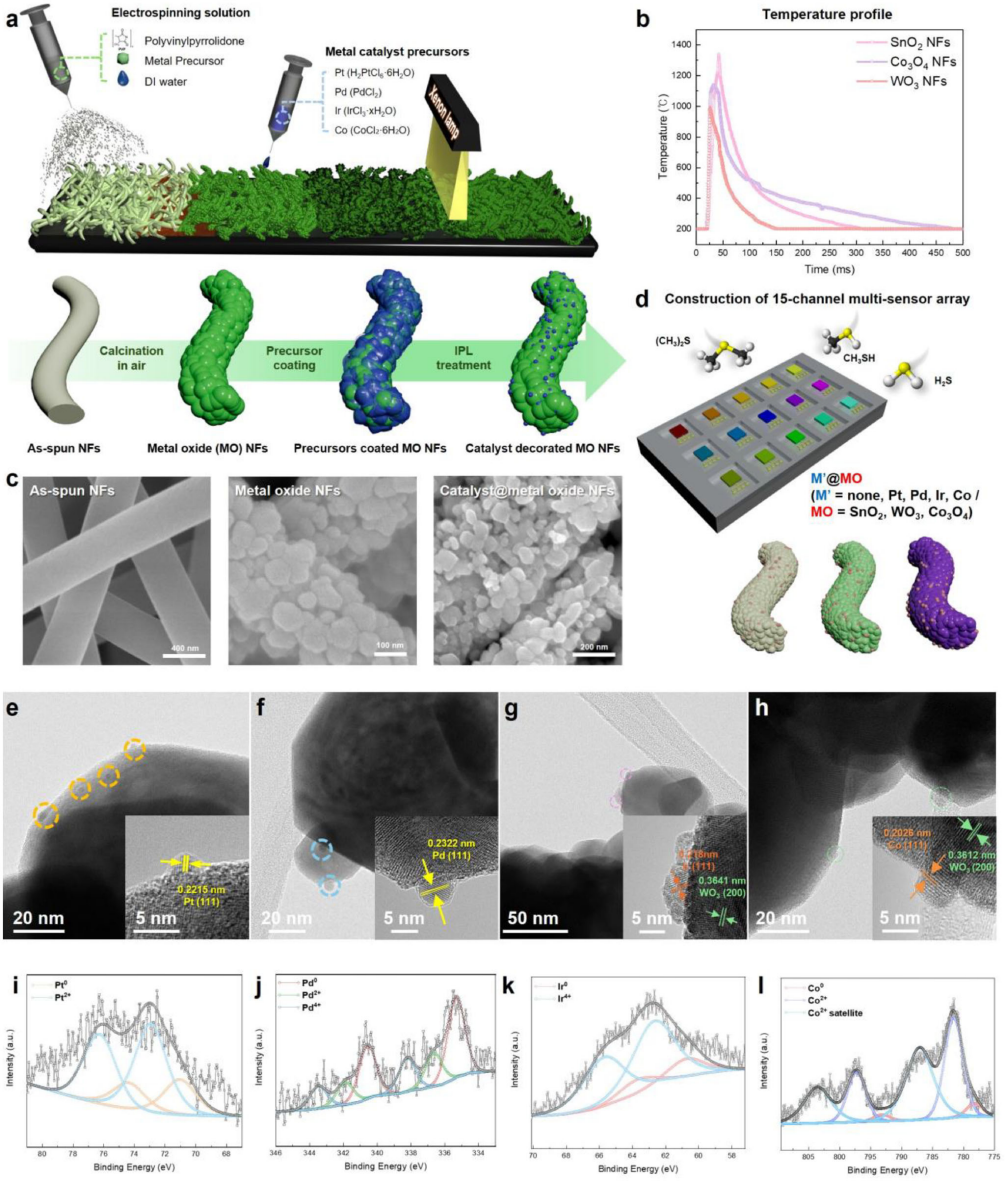
Overview of photothermally synthesized metal‐catalyst‐decorated metal oxide nanofibers and their sensor array integration. (a) Overall schematic of the synthesis of metal oxide NFs vis an electrospinning process followed by IPL‐induced catalytic NPs decoration on the surface of metal oxide NFs under ambient air. (b) Real‐time temperature evolution of metal oxide NFs during IPL irradiation, recorded by an infrared camera. (c) SEM images of as‐spun composite NFs, after calcination, and after IPL treatment. (d) Conceptual illustration of fifteen distinct sensor array for the detection of VSCs gas species. High‐resolution TEM images of (e) Pt@WO_3_, (f) Pd@WO_3_, (g) Ir@WO_3_, and (h) Co@WO_3_ NFs, confirming the formation of uniformly distributed metallic NPs on the oxide surface after the IPL process. XPS spectra of (i) Pt 4f, (j) Pd 3d, (k) Ir 4f, and (l) Co 2p regions after IPL treatment, revealing the photothermal reduction of metal precursor species into mixed metallic/oxidized states.

Catalytic NPs decorated with metal oxide NFs were fabricated via an electrospinning process followed by subsequent calcination and IPL‐assisted photothermal process (Figure 1a and Figure S1). First, pure metal oxide NFs were synthesized by electrospinning a precursor solution composed of metal oxide precursors (Sn(Oct)_2_, Co(CH_3_COO)_2_·4H_2_O, or (NH_4_)_6_H_2_W_12_O_40_·xH_2_O), polymer (polyvinylpyrrolidone, PVP), and deionized water, followed by calcination in air. Because each metal oxide requires a different crystallization temperature, calcination was performed at 500, 400, and 600°C for SnO_2_, Co_3_O_4_, and WO_3_ NFs, respectively (Figure S2). Then, catalytic metal precursor solutions prepared by dissolving the corresponding metal salts (H_2_PtCl_6_, PdCl_2_, IrCl_3_, or CoCl_2_) in ethanol were drop‐cast onto the as‐prepared metal oxide NFs, followed by IPL treatment. During the 20‐millisecond irradiation with IPL at an energy density of 18.698 J/m^2^, rapid photothermal heating raised the local temperature up to ∼1350°C (Figure 1b and Figure S3), which was sufficient to thermally decompose and reduce the precursors, forming ultra‐small catalytic metal NPs uniformly anchored on metal oxide NFs (Figure 1c). Hereafter, catalytic metal NPs anchored metal oxide NFs are denoted as M@SnO_2_, M@Co_3_O_4_, and M@WO_3_, where M represents Pt, Pd, Ir, and Co. Based on these fifteen M@metal oxide NF compositions, we fabricated fifteen distinct sensor elements by drop‐coating each material onto individual sensor substrates (Figure 1d).

High‐resolution transmission electron microscopy (HRTEM) images confirm the formation of sub‐10 nm metal NPs that are uniformly distributed along the WO_3_ NFs after IPL treatment (Figure S4). Each catalyst type (Pt, Pd, Ir, and Co) forms well‐defined crystalline NPs anchored to the oxide surface, as evidenced by clear lattice fringes and distinct particle boundaries (Figure 1e–h). The uniformly dispersed NPs observed across all four metals highlight the rapid nucleation and surface‐localized reduction enabled by the IPL process. Given that the IPL treatment is conducted under ambient air conditions, partial oxidation of the NP surface is inevitable. X‐ray photoelectron spectroscopy (XPS) analyses confirmed the partial oxidation of each metal, revealing the coexistence of metallic species (Pt^0^, Pd^0^, Ir^0^, Co^0^) and their corresponding oxidized states (Pt^2+^, Pd^2+^/Pd^4+^, Ir^4+^, Co^2+^) (Figure 1i–l) [6]. Although the samples were prepared using identical weight ratios of metal catalyst precursors, the final metal compositions (wt.%) exhibit some deviations depending on the precursors type, likely due to differences in their thermal decomposition temperatures (Table S1) [18, 19, 20, 21, 22, 23, 24, 25].

X‐ray diffraction (XRD) analysis further confirmed that the crystallographic structures of WO_3_, SnO_2_, and Co_3_O_4_ NFs remained unchanged after the IPL‐induced photothermal process, without noticeable peak shifts or phase transitions (Figure S5). Diffraction peaks corresponding to Pt, Pd, Ir, or Co were not observed, which is attributed to their extremely low loading amounts (≤ 0.01 wt.%) and nanoscale particle sizes falling below the XRD detection limit (Figure S5a). Additionally, XPS of the W 4f spectra revealed that the relative W^6+^/W^5+^ ratio in WO_3_ exhibited no appreciable change before and after IPL treatment, indicating that the ultrafast high‐temperature exposure does not induce significant oxygen vacancy formation or lattice reduction (Figure S6). These results collectively suggest that while the momentary photothermal shock is sufficient to rapidly nucleate and anchor catalytic metal NPs, it does not compromise the structural integrity or chemical stability of the underlying metal oxide‐based sensing layers [6, 10, 16, 17].

### 2.2 Chemiresistive Gas Sensing Performances

**FIGURE 2:**
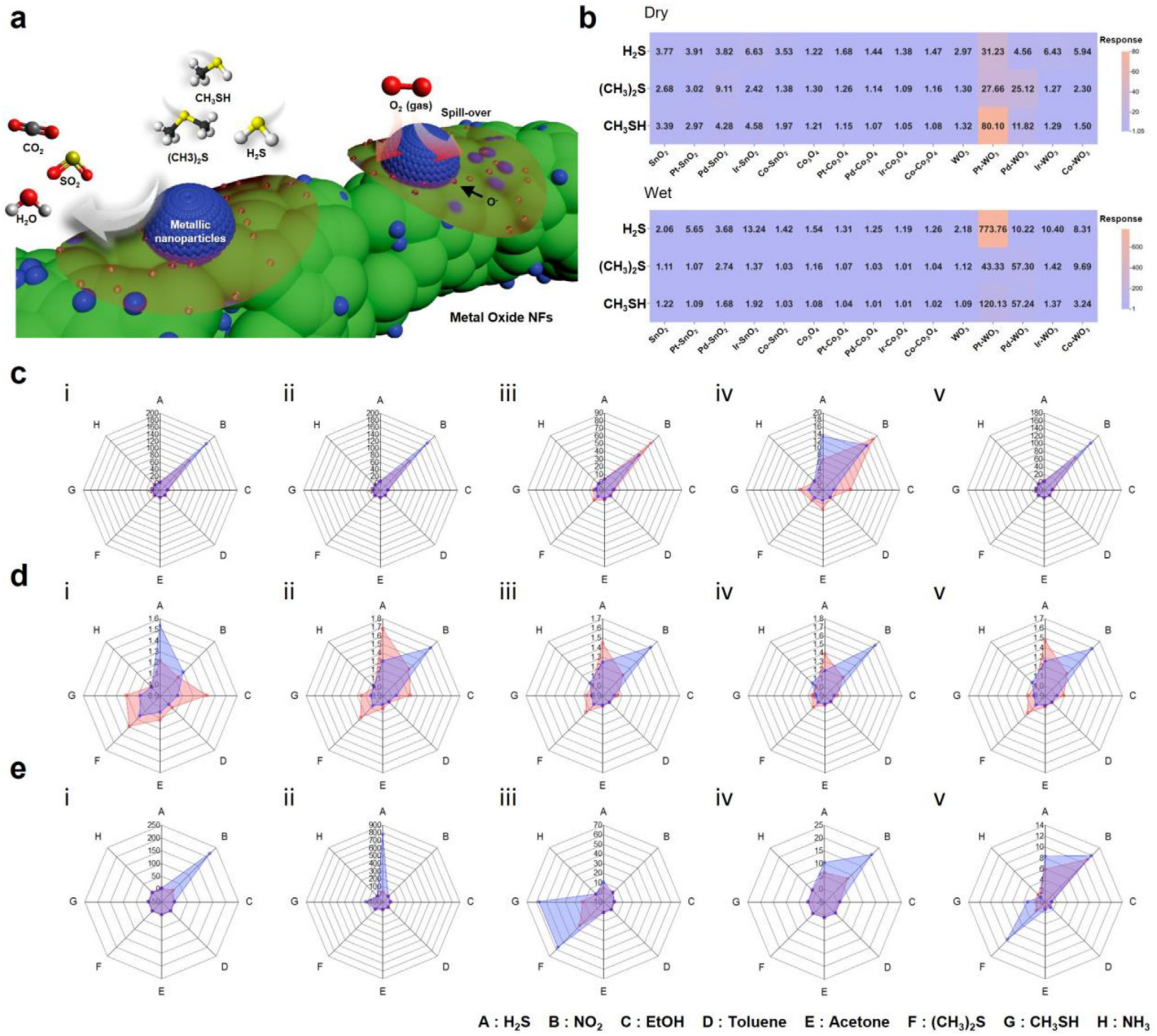
Reaction mechanism and selective sensing behaviors of 15 sensor array toward VSCs. (a) Schematic illustration of the catalytic oxidation mechanism, in which VSCs are adsorbed and oxidized on metal‐decorated metal oxide NFs surfaces, producing SO_2_, H_2_O, and CO_2_ as byproducts. (b) Normalized response heatmaps of various metal–oxide NFs showing distinct reaction patterns for 1 ppm of each sulfur compound, confirming the role of catalytic metals (Pt, Pd, Ir, Co) in modulating gas–surface interactions. (c–e) Radar plots comparing the gas‐response distribution of pristine and catalyst (Pt, Pd, Ir, Co)‐decorated metal oxide NFs sensor arrays toward 1 ppm of 8 gas analytes, illustrating the enhanced selectivity and response magnitude achieved by metal incorporation: (c) SnO_2_, (d) Co_3_O_4_, (e) WO_3_ NFs. Red regions represent sensing responses measured under dry conditions (0% RH), whereas blue regions represent measurements conducted under humid conditions (90% RH).

The gas‐sensing characteristics of the M@SnO_2_, M@Co_3_O_4_, and M@WO_3_ (M = none, Pt, Pd, Ir, and Co) were investigated toward three representative VSCs, including hydrogen sulfide (H_2_S), methyl mercaptan (CH_3_SH), and dimethyl sulfide ((CH_3_)_2_S), as well as five potential interfering gas analytes, including nitrogen dioxide (NO_2_), ethanol (C_2_H_5_OH), toluene (C_7_H_8_), acetone (CH_3_COCH_3_), and ammonia (NH_3_) (**Figure** **2**). First, given that metal oxide gas sensors require thermal activation, we characterized the 15‐channel sensor array by exposing it to 1 ppm gas analytes across four operating temperatures (200, 240, 290, and 340°C) (Figures S7 and S8). As a representative sensor, Pt@WO_3_ NFs exhibited the highest H_2_S response at 240°C compared to lower (200°C) or higher temperatures (290 and 340°C) with reliable reversibility. Accordingly, 240°C was carefully selected as the optimized operating temperature for subsequent detailed sensing analyses using 15‐channel sensor array platform.

Figure 2a presents a schematic illustration of the sensing mechanisms in catalyst‐decorated metal oxide NFs, highlighting the catalytic oxidation of sulfur molecules, i.e., H_2_S, CH_3_SH, (CH_3_)_2_S, on the metallic NPs. These activated sulfur‐containing fragments subsequently spill‐over onto the oxide surface, where they react predominantly with chemisorbed oxygen species (O^–^, O_2_^–^) [26, 27]. This oxidation process produces SO_2_, CO_2_, and H_2_O while releasing electrons into the oxide conduction band [28]. The overall reaction pathways for H_2_S, CH_3_SH, and (CH_3_)_2_S follow the commonly accepted chemisorbed‐oxygen oxidation model, as summarized below.

(1) $$H_{2}S\;\left(\mathrm{gas}\right)+3O^{-}={\mathrm{SO}}_{2}+H_{2}O+3e^{-}$$

CH_3_SH (gas)  +  6O^−^  =  SO_2_  +  2H_2_O  +  CO_2_  +  6e^−^
: (2)

(3) $${(CH_{3})}_{2}S\;\left(\mathrm{gas}\right)+9O^{-}=SO_{2}+3H_{2}O+2CO_{2}+9e^{-}$$

These reaction pathways collectively indicate that the sensing characteristics of VSCs can be systematically tuned by varying the catalyst‐oxide combination, reflecting the strong influence of catalytic activation pathways and spill‐over mediated oxidation kinetics. Detection of trace‐level VSCs (100–1000 ppb) is important for halitosis‐related breath monitoring, as the relative abundance of VSCs has been associated with oral malodor severity [29].

Since exhaled breath contains a high level of humidity, humidity‐controlled measurements were conducted to examine the sensing behavior under a more realistic background. Accordingly, a bubbler system was used to generate 90% relative humidity (90% RH), approximating the humidity level inside the oral cavity, and additional sensor measurements were performed under these conditions (Figures S9–S11). This hydroxyl‐assisted oxidation pathway modulates the surface reactivity of several catalyst‐oxide combinations [30], resulting in humidity‐dependent response variations across the measured concentration range of 100–1000 ppb rather than uniformly enhanced responses under humid conditions (Figure 2b and Figure S12). To further assess trace‐level VSC sensing under breath‐relevant humidity, additional measurements at 50 and 25 ppb under 90% RH confirmed that the 15‐channel sensor array retained discernible response profiles toward H_2_S, CH_3_SH, and (CH_3_)_2_S (Figure S13). Additionally, Figure 2c–e presents how catalyst‐oxide combinations shape the selectivity landscape of the 15‐channel sensor array toward eight gases at 1 ppm under both dry (0% RH, red) and humid (90% RH, blue) conditions. For example, pristine SnO_2_ NFs, Pt@SnO_2_ NFs, and Co@SnO_2_ NFs channels exhibit stronger responses toward NO_2_ under humid conditions (90% RH) than under dry air, indicating enhanced reactivity in moisture‐rich environments (Figure 2c). In contrast, p‐type Co_3_O_4_ exhibits relatively low response amplitudes, yet the catalyst‐decorated channels display clearer NO_2_ selectivity under humid conditions (90% RH) than in dry air (Figure 2d). Meanwhile, WO_3_ shows the most pronounced catalyst‐induced response separation, with Pt‐, Pd‐, and Co‐functionalized sensors generating clearly defined response lobes toward the three sulfur compounds (Figure 2e). To further examine the response of the sensor array to coexisting VSCs in complex sulfur‐containing gas environments, additional mixed‐gas sensing experiments were conducted using binary and ternary sulfur‐compound mixtures under dry and humid conditions. Each VSC component was introduced at 1 ppm, corresponding to total VSC concentrations of 2 ppm for the binary mixtures and 3 ppm for the ternary mixture (Figures S14–S16). The results show that the 15‐channel sensor array generates clear catalyst‐ and oxide‐dependent resistance modulations and composition‐specific sensing fingerprints across single, binary, and ternary VSC conditions.

Although the absolute response amplitudes vary among the three different metal oxides when transitioning from dry to humid environments, the characteristic selectivity patterns of each catalyst‐metal oxide pair are largely preserved, and in some cases become more distinguishable at 90% RH. Such behavior indicates that moisture‐driven hydroxyl chemistry can partially promote gas‐surface interactions [31, 32, 33]. In addition to response magnitude and selectivity, the dynamic sensing characteristics were further evaluated by analyzing the response and recovery times toward individual sulfur gases, including H_2_S, CH_3_SH, and (CH_3_)_2_S, at 1 ppm (Figure S17).

To further examine the sensing behavior under a breath‐derived background, complementary breath‐matrix measurements were conducted using exhaled breath collected in a Tedlar bag (Figure S18). As shown in Figure S19, resistance profiles and response values were obtained under exhaled breath alone, exhaled breath with 1 ppm H_2_S, exhaled breath with 1 ppm CH_3_SH, and exhaled breath with their binary mixture. Although exhaled breath alone induced noticeable resistance variation, sulfur‐compound introduction produced additional VSC‐dependent response profiles, providing preliminary support for the potential applicability of the WO_3_‐based sensor group to breath‐matrix VSC analysis. The humidity stability and reproducible sensing behavior of the 15‐channel sensor array were experimentally confirmed under both dry and humid atmospheres. Across 20 repeated exposure‐recovery cycles conducted under both dry (0% RH) and humid (90% RH) conditions, each sensor channel maintained stable response magnitudes and highly consistent curve shapes (Figure S20). This reproducible behavior confirms that the IPL engineered catalyst‐oxide interfaces are sufficiently robust and enable reliable performance suitable for repeated operation in moisture‐containing environments.

Although the three sulfur‐containing gases and their mixtures exhibit distinguishable response patterns across the 15‐channel sensor array (Figure 2b, Figure S16, and Table S2), their similar surface‐oxidation pathways and partially overlapping response behaviors make fully reliable discrimination challenging when relying solely on simple response amplitudes or individual resistance profiles. Moreover, practical breath‐related sensing studies require not only identifying the gas species and estimating their concentration but also quantifying uncertainty to improve prediction reliability. To address these challenges, we employ a deep‐ensemble Transformer model that leverages the full multichannel transient dataset from the 15‐channel sensor array. By capturing subtle temporal dynamics and inter‐sensor correlations beyond simple amplitude comparison, this framework enables simultaneous gas‐species classification, concentration regression, and explicit uncertainty quantification.

## 3 Reliability‐Aware Deep Learning for Selective Sulfur Gas Identification and Quantification

### 3.1 Input Data and Preprocessing

We use multichannel time series from a 15‐channel catalyst–metal oxide NFs sensor array, exposed to 8 analyte gases under single‐gas and gas‐mixture conditions with multiple concentrations (100, 400, 700, 1000 ppb) and humidity levels (dry and 90% RH) with five repeated measurements for each condition. Each sensing experiment yields a continuous 35 min recording of all 15 resistance channels. From these recordings, we extract overlapping fixed‐length windows of length T = 40 time steps with sampling interval ∆t = 0.5 min, (corresponding to 20 min per window), using a stride of 0.5 min between consecutive windows (Table S3).

For each window, the model jointly predicts gas‐wise concentrations and multi‐label gas identities. Specifically, for *C*  =  8 target gases, the regression target is represented as a concentration vector $\rho \;=[\rho_{1},\rho_{2},\text{…},\rho_{C}]$, and the classification target is represented as a multi‐hot label vector $z=[z_{1},z_{2},\text{…},z_{C}]$, where *z_c_* =  1 indicates the presence of gas *c* and *z_c_* =  0 otherwise. This formulation allows multiple gas species to be simultaneously present in a single window. To stabilize the regression, each gas‐wise concentration target is standardized as

(4) $$y_{c}=\frac{\left(\rho_{c}\;-\;\mu_{\rho ,c}\right)}{\sigma_{\rho ,c}},\;c=1,\text{…},C,$$

where µ_ρ,*c*_, σ_ρ,*c*_ are the mean and standard deviation of the concentration of gas *c* over the training set.

**FIGURE 3:**
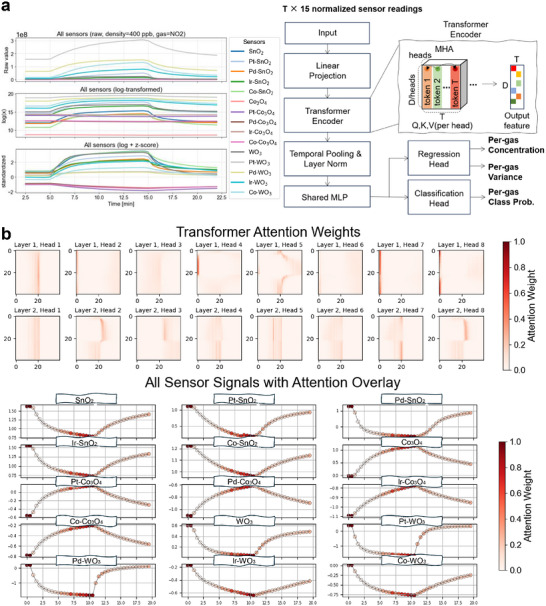
Input preprocessing, Transformer‐based architecture, and attention visualization. (a) Representative 20‐min window of the 15‐channel sensor array showing raw resistance traces (top), log‐transformed responses (middle), and standardized log‐responses after z‐score normalization (bottom), together with the overall Transformer‐based multi‐task architecture that maps the T ×15 normalized sensor readings to per‐gas concentration estimates, per‐gas predictive variances, and per‐gas class probabilities. Within the Transformer encoder block (right), multi‐head self‐attention is illustrated: for each of the 8 heads, query, key, and value vectors of dimension D/8 are computed at each of the T time steps, attention is applied over the temporal dimension within each head, and the resulting head outputs are concatenated and projected back to T × D tokens. (b) Attention‐weight visualization for a representative test sample. Top: self‐attention heatmaps for all heads in the two encoder layers; bottom: time series of all 15 sensor channels with attention weights overlaid as color intensity, providing a qualitative visualization of temporal regions attended by the model during joint gas identification and concentration prediction.

The raw resistance signals from the 15 sensors are denoted by R_t,s_ where t = 1, …, T indexes time and s = 1, …, S indexes the sensor channel with S = 15. To reduce dynamic range and improve numerical stability, we first apply a logarithmic transform,

(5) $${\tilde{x}}_{t,s}=\log\left(\max\left(R_{t,s^{\prime }}\varepsilon \right)\right),$$

with a small ε to avoid singularities, and then perform per‐sensor z‐score normalization using training‐set statistics:

(6) $$x_{t,s}=\frac{\left({\tilde{x}}_{t,s}\;-\;\mu^{\left(s\right)}\right)}{\;\sigma^{\left(s\right)}},$$

where  µ^(*s*)^ and  σ^(*s*)^ denote the mean and standard deviation of ${\tilde{x}}_{t,s}$ over all windows in the training set for sensor s. The resulting input window is represented as a matrix X ∈ $R^{T\times S}$ that serves as input to the deep learning model. A representative example of the raw, log‐transformed, and standardized sensor responses over a 20 min window is shown in Figure 3, left panels.

### 3.2 Transformer‐Based Multi‐Task Architecture

To endow the chemically engineered sensor array with reliability‐aware decision‐making capability, we adopt a lightweight Transformer‐based multi‐task architecture that maps each input window *X* ∈ $R^{T\times S}$ to a shared latent representation, followed by task‐specific heads for gas‐wise concentration regression, gas‐wise predictive variance estimation, and multi‐label gas identity classification. Compared to recurrent architectures, the self‐attention mechanism enables the model to capture temporal dependencies in gas‐response dynamics and provides qualitative attention visualizations of temporal regions used by the model. An overview of the input preprocessing (time‐series panels) and the Transformer‐based multi‐task architecture is provided in Figure 3, right. The S‐dimensional sensor readings at each time step are first projected to a d‐dimensional embedding space using a learned linear projection, and a learnable positional embedding is added to encode temporal order:

(7) $$Z_{0}=\mathrm{Linear}\left(X\right)+P\in R^{T\times d},d=128,$$

where $P\in R^{T\times d}$ denotes a learned positional embedding. This representation can be interpreted as a low‐dimensional, temporally ordered embedding of the multichannel sensor array at each time step. To capture temporal dependencies and inter‐sensor correlations, we stack two Transformer encoder layers with pre‐norm residual connections and dropout. At layer *l*, given input *H* ^(*l*)^ ∈ $R^{T\times d}$, the self‐attention and feed‐forward sublayers are defined as

(8) $$\mathrm{SA}\left(H^{\left(l\right)}\right)=\mathrm{MHA}\left(H^{\left(l\right)},H^{\left(l\right)},H^{\left(l\right)}\right),$$

(9) $$\mathrm{FFN}\left(H^{\left(l\right)}\right)=\mathrm{Linea}r_{2}\left(\mathrm{ReLU}\left(\mathrm{Linea}r_{1}\left(H^{\left(l\right)}\right)\right)\right)$$

where *MHA* denotes multi‐head self‐attention [34] with 8 heads. Each encoder layer uses the standard pre‐norm residual structure with dropout 0.2:

(10) $${\tilde{H}}^{\left(l\right)}=H^{\left(l\right)}+\mathrm{Drop}\mathrm{out}\left(\mathrm{SA}\left(\mathrm{LN}\left(H^{\left(l\right)}\right)\right)\right),$$

(11) $$H^{\left(l+1\right)}={\tilde{H}}^{\left(l\right)}+\mathrm{Drop}\mathrm{out}\left(\mathrm{FFN}\left(\mathrm{LN}\left({\tilde{H}}^{\left(l\right)}\right)\right)\right),$$

with layer normalization *LN*(·) applied before each sublayer [35]. We record the per‐head attention weight matrices in each encoder layer for qualitative analysis, but they are not used directly in the loss.

Concretely, given *H* ^(*l*)^ ∈ $R^{T\times d}$, the multi‐head self‐attention module first computes, for each of the h = 8 heads, query, key, and value matrices *Q_i_*, *K_i_*, and *V_i_* ∈ $R^{T\times d_{h}}$, where *d_h_* = d / h. Scaled dot‐product attention is then applied over the T time steps within each head, and the resulting per‐head representations *Attn_i_* (*H* ^(*l*)^) ∈ $R^{T\times d_{h}}$ are concatenated along the feature dimension and linearly projected back to $R^{T\times d}$. The output of the final encoder layer Z ∈ $R^{T\times d}$ is aggregated along the temporal dimension via mean pooling, followed by layer normalization.

(12) $$\bar{z}=\mathrm{LN}\left(\frac{1}{T}\sum_{t=1}^{T}Z_{t}\right)\in R^{d}.$$

This pooled representation summarizes the time window and is passed through a shared multilayer perceptron (MLP),

(13) $$h=MLP_{shared}\;\left(\bar{z}\right)\in R^{64},$$

where the *MLP* consists of fully connected layers with *ReLU* nonlinearities and dropout. From the shared representation *h*, we branch into regression and classification heads. The regression head outputs gas‐wise predictive means and raw scale parameters, $\left(\mu ,\tilde{s}\right)\in R^{C}\times R^{C}$, where µ_*c*_ represents the predicted standardized concentration of gas *c*, and ${\tilde{s}}_{c}$ is mapped to a positive gas‐wise predictive variance $\sigma_{c}^{2}$. The classification head outputs gas‐wise logits $o\in R^{C}$, and the corresponding gas‐presence probabilities are computed independently using a sigmoid function, p  =  σ(o). This multi‐label formulation allows multiple gas classes to be simultaneously activated in mixture samples.

To interpret how the Transformer encoder uses temporal information, we visualize the learned self‐attention patterns. For a representative test sample, we plot per‐head attention maps for both encoder layers and overlay the attention weights on the 15‐channel sensor traces (Figure 3b). These attention maps are used as qualitative visualizations of model behavior and indicate temporal regions attended by the model, without implying causal feature attribution or sensor‐channel importance.

### 3.3 Deep Ensemble and Uncertainty Modeling

**FIGURE 4:**
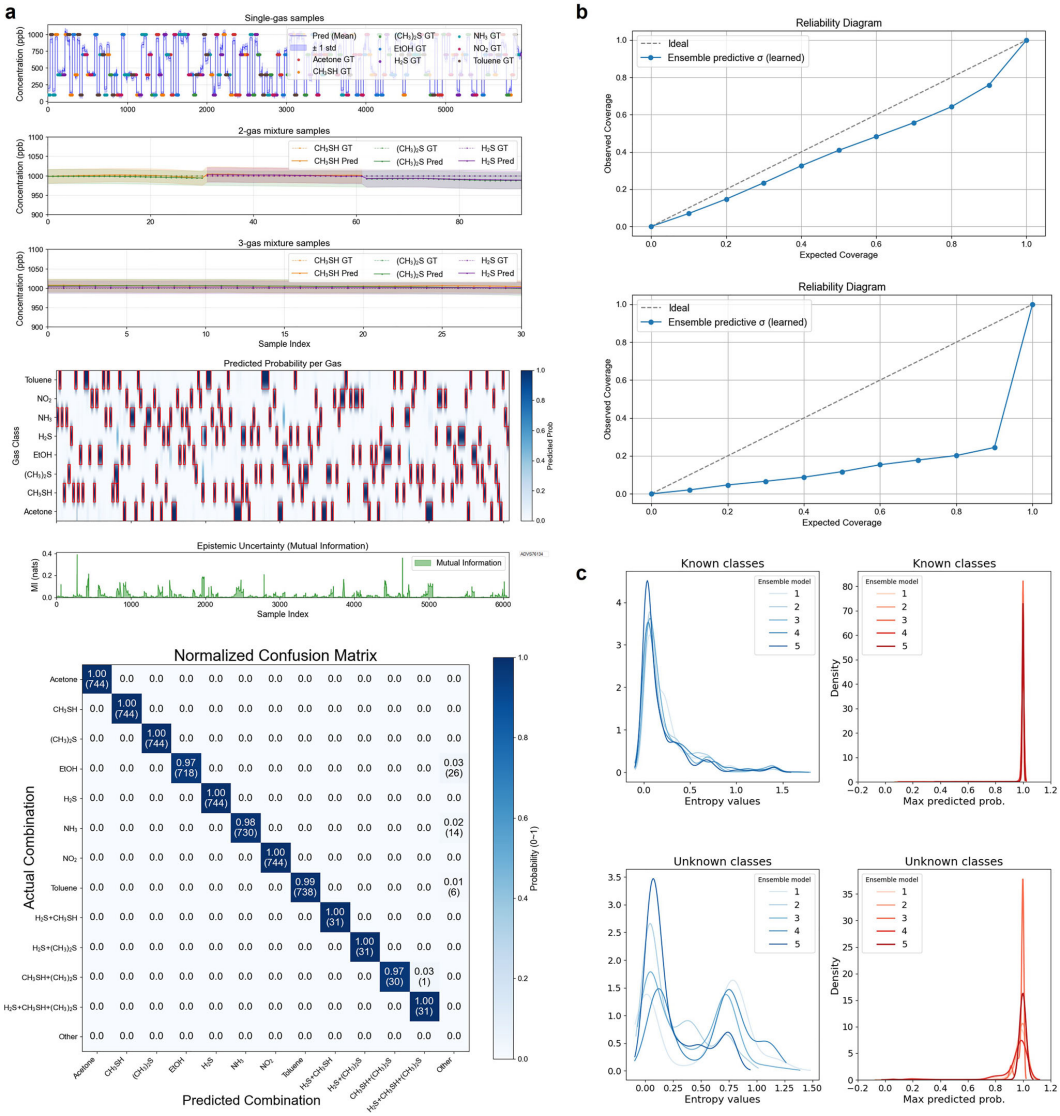
Comprehensive evaluation of multi‐gas identification, concentration prediction, calibration, and uncertainty estimation. (a) Representative model outputs for single‐gas, two‐gas mixture, and three‐gas mixture samples. The upper plots compare ground‐truth and predicted gas concentrations, with shaded regions indicating one standard deviation of the predicted concentration. The probability heatmap shows the predicted probability for each gas class, where red borders indicate the ground‐truth gas labels. The mutual information (MI) plot represents epistemic uncertainty estimated from the deep ensemble. The normalized confusion matrix summarizes the classification performance for single‐gas and gas‐mixture combinations. (b) Calibration analysis of regression uncertainty: reliability diagrams comparing expected and observed coverage of Gaussian prediction intervals obtained from the learned ensemble predictive variance (upper) and from an empirical baseline that uses only the variance of ensemble means (“µ‐variance”, lower). The deep‐ensemble predictive variance yields substantially better calibrated intervals than the empirical baseline. Overall, this demonstrates that the reported predictive intervals and confidence scores can be interpreted as meaningful measures of risk, which is essential if uncertainty is to be used for tasks such as reliability‐aware thresholding, OOD detection, or triggering confirmatory measurements in practical e‐nose systems. (c) Kernel density estimates of per‐model class‐wise Bernoulli entropy (blue) and maximum gas‐presence probability (red) for in‐distribution (known) test gases (left) and an out‐of‐distribution evaluation set (right), showing higher entropy and lower confidence for unknown samples.

To improve robustness and quantify uncertainty, we adopt a deep ensemble [36] of *M* = 5 independently initialized models with identical architecture and training hyperparameters. At inference time, we aggregate predictions across ensemble members and decompose uncertainty into aleatoric (data noise) and epistemic (model) components for both regression and classification. Figure 4a illustrates example test‐set predictions (Figures S21 and S22): the top panel shows ensemble mean regression outputs with ±1σ predictive bands, the bottom panel shows classification outputs together with epistemic uncertainty quantified by mutual information, and the rightmost subpanel displays the normalized confusion matrix.

For the regression task, each model predicts gas‐wise standardized concentration targets $y\;=[y_{1},\text{…},y_{C}]$. For each gas class *c*, the target *y_c_* is modeled with a Gaussian likelihood.

(14) $$y_{c}∼N\left(\mu_{c},{\sigma_{c}}^{2}\right),\;c=1,\text{…},C,$$

where µ_*c*_ is the predicted standardized concentration of gas *c*, and the gas‐wise variance $\sigma_{c}^{2}$ is parameterized via a positive mapping of the raw scale ${\tilde{s}}_{c}$:

(15) $${\sigma_{c}}^{2}=\tau \left(softplus\left({\tilde{s}}_{c}\right)+\varepsilon \right),c=1,\text{…},C,$$

with ε = 10^−6^  and scaling factor τ = 1 unless otherwise stated. For each gas class, the heteroscedastic negative log‐likelihood (NLL) is computed as

(16) $$L_{reg,c}=\frac{1}{2}\;\left(\log\left({\sigma_{c}}^{2}\right)+\left(\frac{{\left(y_{c}-\mu_{c}\right)}^{2}}{{\sigma_{c}}^{2}}\right)\right),\;c=1,\text{…},C.$$

Since each sample contains only a subset of the target gases, the regression loss is computed only for active gas classes using the multi‐hot gas‐presence label as a mask. Thus, concentration errors are penalized only for gases present in each sample, while absent gas classes are excluded from the regression loss. This masked heteroscedastic formulation allows the model to express higher variance in regions of the input space with intrinsically noisy or ambiguous signals [37].

For the classification task, each model outputs logits *o* ∈ $R^{C}$ which are converted into gas‐presence probabilities using a sigmoid function, p = sigmoid(o). Given the multi‐hot ground‐truth label vector $z\in {\left\{0,1\right\}}^{C}$, where *z_c_* = 1 indicates the presence of gas *c*, we use the binary cross‐entropy loss for multi‐label classification:

(17) $$L_{cls}=BCEWithLogits\left(o,z\right)$$

In the main experiments, the total loss is defined as a weighted sum of the masked heteroscedastic regression loss and the multi‐label classification loss:

(18) $$L=L_{\mathrm{reg}+}w_{\mathrm{cls}}L_{\mathrm{cls}}.$$

Let µ_*m*,*c*_ and σ_*m*,*c*_^2^ denote the mean and variance predicted by ensemble member m for gas class *c*. The ensemble predictive mean and variance are computed for each gas class as

(19) $$E\left[y_{c}\right]\approx \;{\bar{\mu }}_{c}=\frac{1}{M}\;\sum_{m=1}^{M}\mu_{m,c},$$

(20) $$Var\left[y_{c}\right]\approx \frac{1}{M}\sum_{m=1}^{M}{\sigma_{m,c}}^{2}+Var_{m}\left(\mu_{m,c}\right),$$

where the first term represents gas‐wise aleatoric uncertainty and the second term represents gas‐wise epistemic uncertainty.

For classification, each model produces gas‐wise probabilities using a sigmoid activation, **p**_*m*_ = sigmoid(**o**_*m*_). We average them across ensemble members to obtain the ensemble gas‐presence probabilities:

(21) $$\bar{p}=\frac{1}{M}\;\sum_{m=1}^{M}p_{m}.$$

Since the task is formulated as multi‐label classification, predictive uncertainty is computed using class‐wise Bernoulli entropy. For gas class *c*, the entropy of the ensemble prediction is

(22) $$H\;\left({\bar{p}}_{c}\right)=-{\bar{p}}_{c}\log\left({\bar{p}}_{c}\right)-\left(1-{\bar{p}}_{c}\right)\log\left(1-{\bar{p}}_{c}\right).$$

The epistemic uncertainty for each gas class is quantified by mutual information:

(23) $$MI_{c}=H\left({\bar{p}}_{c}\right)-\frac{1}{M}\sum_{m=1}^{M}\left(H\left(p_{m,c}\right)\right).$$

The total entropy and MI can be summarized by averaging or summing the class‐wise values across gas classes.

In summary, the regression head provides an explicit decomposition of predictive uncertainty into aleatoric and epistemic terms through the ensemble variance formula: the first term in *Var*[*y_c_*] averages the per‐model variances σ^2^_*m*,*c*_ and captures input‐dependent data noise (heteroscedastic aleatoric uncertainty), while the second term *Var_m_* (µ_*m*,*c*_) reflects disagreement between ensemble members and thus corresponds to epistemic, or model, uncertainty. For the classification head, each network outputs gas‐wise presence probabilities without an explicit variance parameter, so we interpret the class‐wise Bernoulli entropy as total uncertainty (aleatoric + epistemic), and the mutual information *MI* as the epistemic component arising from model‐to‐model disagreement. This ensemble‐based treatment allows us to use a single multi‐task architecture while still distinguishing between data noise and model uncertainty across both gas‐wise regression and multi‐label classification outputs.

In prospective applications, these uncertainty estimates could be exploited in several ways: for instance, by rejecting low‐confidence predictions to trade coverage for accuracy, by flagging high class‐wise entropy or high‐MI samples as candidates for out‐of‐distribution (OOD) or sensor‐fault detection, or by prioritizing high‐uncertainty conditions for additional measurements or re‐training in an active‐learning loop.

### 3.4 Calibration of Predictive Uncertainty

We explicitly assess whether the predicted uncertainties are calibrated. For regression, we treat the ensemble predictive mean and standard deviation, (${\bar{\mu }}_{c}$, ${\bar{\sigma }}_{c}$), as defining a gas‐wise Gaussian predictive distribution for each gas class *c*. For a range of nominal coverage levels, we construct prediction intervals [${\bar{\mu }}_{c}$ ± $z_{\alpha }{\bar{\sigma }}_{c}$], where z_α_ is the corresponding Gaussian quantile, and compare the empirical fraction of active gas‐wise test targets whose true concentrations fall inside these intervals with the nominal coverage, yielding a regression reliability diagram. Figure 4b summarizes these results: the left panel shows that the coverage of intervals built from the ensemble predictive variance closely follows the ideal identity line, while the right panel compares this to a baseline that uses an empirical standard deviation derived only from the variance of ensemble means (µ‐variance), which consistently underestimates the true uncertainty and therefore exhibits noticeably poorer calibration.

Intuitively, a well‐calibrated model is one whose stated uncertainty matches empirical error frequencies: among all active gas‐wise test samples assigned a nominal coverage level, approximately the same fraction of true concentrations should fall inside the corresponding prediction interval. This property is crucial if the predicted uncertainty is to be used as a principled confidence measure for downstream decision‐making.

Beyond average metrics, we also examine how the ensemble discriminates between in‐distribution and OOD gases. As shown in Figure 4c, OOD evaluation samples exhibit substantially higher class‐wise Bernoulli entropy and lower maximum gas‐presence probabilities than known test gases, indicating that the ensemble assigns lower confidence to previously unseen analytes.

### 3.5 Training and Evaluation Protocol

To avoid same‐run data leakage, all train/validation/test splits are performed at the level of entire sensing runs. For each sensing scenario, five independent experimental runs were acquired, and one run (20%) was reserved exclusively for testing, while the remaining runs were used for training and validation. All models are trained using the Adam optimizer with an initial learning rate of 3 × 10^−4^, a batch size of 16, and a maximum of 30 training epochs. Input features are normalized using statistics computed from the training set, and the regression target corresponds to gas‐wise normalized concentrations. All reported test results are obtained from a single evaluation per random seed; ensemble metrics are computed by aggregating predictions across *M* = 5 independently trained members, as described in Section “Deep Ensemble and Uncertainty Modeling”.

To ensure a fair comparison across architectures, all individual models are configured with a comparable number of trainable parameters: the MLP baseline uses approximately 303K parameters, the LSTM baseline approximately 299K parameters, and each Transformer model approximately 292K parameters. The deep ensemble consists of *M* = 5 independently trained Transformer models, each with the same architecture and parameter count. This design isolates the impact of architectural differences and ensemble learning from per‐model capacity effects.

For the regression task, Table 1 summarizes the quantitative comparison across models using mean absolute error (MAE) and root mean squared error (RMSE), both reported in the original concentration space. Among all evaluated architectures, the proposed deep ensemble Transformer achieves the best regression performance, reducing MAE from 0.053 ppm to 0.045 ppm and RMSE from 0.075 ppm to 0.062 ppm compared to the single Transformer. These improvements highlight the benefit of ensemble averaging and uncertainty‐aware training in reducing concentration prediction error. In addition to pointwise accuracy, uncertainty quality is further assessed using regression calibration curves, as shown in Figure 4b, and representative uncertainty‐aware total concentration regression results are provided (Figure S23).

**TABLE 1:** Overall regression and classification performance of baseline models and the proposed deep‐ensemble Transformer.

Model	Regression		Classification			
	MAE (ppm)	RMSE (ppm)	Acc (%)	F1‐micro (%)	NLL_cls_ (‐)	Brier (‐)
**MLP baseline**	0.076	0.100	87.08	93.32	0.0380	0.0120
**LSTM baseline**	0.139	0.171	96.15	98.16	0.0178	0.0045
**Single Transformer**	0.053	0.075	98.44	98.99	0.0076	0.0019
**Deep Ensemble** **(proposed)**	**0.045**	**0.062**	**99.23**	**99.62**	**0.0057**	**0.0010**

For the classification task, Table 1 reports overall accuracy, F1‐micro, classification negative log‐likelihood (NLL), and the Brier score for all baseline models and for the proposed deep‐ensemble Transformer, while per‐gas precision/recall/F1 and FP/FN error counts are provided in Tables S4 and S5. Evaluation is conducted on a held‐out test set drawn from the same eight gas classes as the training data. While several models achieve high classification performance, the ensemble consistently yields the most reliable probabilistic predictions. Specifically, the deep‐ensemble Transformer improves accuracy from 98.44% to 99.23% and F1‐micro from 98.99% to 99.62%, while reducing the classification NLL from 0.0076 to 0.0057 and the Brier score from 0.0019 to 0.0010 relative to the single Transformer, indicating improved classification performance, calibration, and reduced overconfidence. Per‐gas concentration prediction and classification results are further provided in Figure S24. To further analyze the behavior of predictive uncertainty, we compare uncertainty statistics under in‐distribution and out‐of‐distribution settings. Figure 4c shows kernel density estimates of the class‐wise Bernoulli entropy and the maximum predicted gas‐presence probability for each ensemble member. The upper panels correspond to the standard test set consisting of the same gas classes used during training (in‐distribution), while the lower panels report results on an unknown‐class evaluation setting. In this setting, the model is trained on only seven gas classes and evaluated on the remaining unseen class, H_2_S. This effect is also reflected in quantitative uncertainty metrics and MI‐based epistemic uncertainty analysis (Figure S25), where the unknown‐class evaluation setting shows increased classification NLL and Brier score compared with the in‐distribution test set. Such an increase indicates that the model assigns lower and more diffuse gas‐presence probabilities to unseen classes, which is desirable behavior when encountering out‐of‐distribution inputs.

Compared to the in‐distribution test samples, the unknown‐class samples exhibit substantially higher class‐wise Bernoulli entropy and lower maximum predicted gas‐presence probability, indicating reduced model confidence. This behavior demonstrates that the deep‐ensemble Transformer appropriately captures epistemic uncertainty and assigns increased uncertainty to samples from previously unseen gas classes, even though they share similar sensing modalities with known classes.

## 4 Conclusion

Reliable chemical sensing requires not only accurate identification of analytes but also quantified confidence in every decision, a capability fundamentally absent in existing electronic‐nose systems. Here, we introduced a reliability‐aware sensing architecture that integrates ultrafast photothermal catalyst engineering with deep‐ensemble learning for volatile sulfur compound (VSC) sensing under breath‐relevant high‐humidity conditions. The momentary IPL process generates chemically diverse catalyst–oxide NFs channels whose heterogeneous reactivity forms a rich response manifold, yet our results reveal that material diversity alone is insufficient to fully resolve the intrinsic overlap among structurally similar sulfur gases. Instead, trustworthy discrimination emerges when this engineered chemical space is coupled with an uncertainty‐calibrated Transformer that can separate aleatoric from epistemic ambiguity, detect out‐of‐distribution events, and assign calibrated confidence intervals aligned with empirical error.

This unified materials‐to‐intelligence framework transforms raw multichannel resistance traces into reliable chemical information, establishing a sensing paradigm where accuracy and confidence are co‐equal requirements. Beyond outperforming standard deep‐learning benchmarks in the identification and quantification of sulfur gases, the explicit uncertainty outputs allow the system to recognize the limits of its own knowledge, a useful capability for future breath‐related and environmental monitoring studies. By demonstrating that quantifiable reliability can be engineered into gas‐sensor arrays through the co‐design of nanoscale catalysts and ensemble AI, this work positions uncertainty quantification as a foundational ingredient of next‐generation intelligent olfaction. Our findings provide a proof‐of‐concept demonstration that gas sensor arrays can report not only predictions but also the reliability of those predictions, which may support future development of practical and trustworthy sensor systems. Further studies involving multiple human subjects, clinically collected breath samples, long‐term sensor drift evaluation, broader VOC/interferent backgrounds, and shorter sensing windows will be required to assess the practical applicability of this platform.

## 5 Experimental Section/Methods

### 5.1 Materials and Reagents

Poly(vinylpyrrolidone) (PVP, Mw ≈ 1,300,000 g/mol), tin(II) 2‐ethylhexanoate (Sn(Oct)_2_, 92.5%), cobalt(II) acetate tetrahydrate (Co(CH_3_COO)_2_·4H_2_O, ≥ 98%), ammonium metatungstate hydrate ((NH_4_)_6_H_2_W_12_O_40_·xH_2_O, ≥ 99%), chloroplatinic acid hexahydrate (H_2_PtCl_6_·6H_2_O, ≥ 99.9%), palladium(II) chloride (PdCl_2_, ≥ 99%), iridium(III) chloride hydrate (IrCl_3_·xH_2_O, ≥ 99%), and cobalt(II) chloride hexahydrate (CoCl_2_·6H_2_O, ≥ 99%) were purchased from Sigma‐Aldrich and used as received without further purification.

### 5.2 Synthesis of Metal Oxide Nanofibers

Three types of metal oxide NFs (SnO_2_, Co_3_O_4_, and WO_3_) were prepared by electrospinning followed by calcination. For SnO_2_ NFs, 1.12 g of Sn(Oct)_2_ and 1.0 g of PVP were dissolved in 12 g of DI water and stirred for 6 h to form a uniform precursor. For Co_3_O_4_ NFs, 3.0 g of Co(CH_3_COO)_2_·4H_2_O and 1.0 g of PVP were dissolved in 8 g of DI water and stirred for 6 h to form a uniform precursor. For WO_3_ NFs, 1.0 g of (NH_4_)_6_H_2_W_12_O_40_·xH_2_O and 1.25 g of PVP were dissolved in 10 g of DI water and stirred for 6 h to form a uniform precursor. Electrospinning was performed using a single‐nozzle system at a tip‐to‐collector distance of 10 cm and a 25 G needle. The applied voltage and flow rate were optimized for each precursor: 13 kV / 10 µL min^−1^ (SnO_2_), 7.5 kV / 25 µL min^−1^ (Co_3_O_4_), and 9 kV / 25 µL min^−1^ (WO_3_). The as‐spun metal oxide precursors/PVP composite NFs were calcined in air for 1 h at 500°C (SnO_2_), 400°C (Co_3_O_4_), and 600°C (WO_3_), respectively, to yield metal oxide NFs (Table S6).

### 5.3 Photothermal Deposition of Metal Nanoparticles

Metal precursor solutions of H_2_PtCl_6_, PdCl_2_, IrCl_3_, and CoCl_2_ (1 mg mL^−1^ in ethanol) were prepared for catalytic nanoparticle functionalization. Then, 50 mg of metal oxide NFs were dispersed in 5 mL of ethanol to form a 10 mg mL^−1^ suspension. A defined amount of metal precursor solution (1 µL) was then introduced into 1 mL of the nanofiber dispersion, resulting in a calculated metal precursor loading of 0.01 wt.% relative to metal oxide NFs. After sonication for 30 min, 10 µL of the mixture was drop‐cast onto glass substrates in each cycle, with a total deposited volume of 60 µL, followed by drying at 60°C for 10 min. The dried films were subjected to single‐shot IPL irradiation (20 ms pulse width, 400 V, energy density = 18.698 J m^−2^, lamp‐to‐sample distance = 2 cm) under ambient air. As a result, metal oxide NFs decorated with photothermally induced catalytic metal NPs were successfully fabricated.

### 5.4 Sensor Fabrication and Gas‐Sensing Measurements

The sensing materials, including pristine and metal‐functionalized SnO_2_, Co_3_O_4_, and WO_3_ NFs (functionalized with Pt, Pd, Ir, or Co), were dispersed in ethanol (6 mg in 300 µL) and sonicated to obtain a uniform ink. Each dispersion was drop‐coated onto an alumina sensor substrate using a micropipette (30 µL per sensor chip) while maintaining the substrate at 60 °C to promote rapid solvent evaporation and uniform adhesion. All alumina sensor chips (2.5 mm × 2.5 mm) were patterned with interdigitated Au electrodes (25 µm width, 70 µm spacing) on the front side and equipped with a Pt microheater on the backside. Two of the four pins were used to measure the resistance across the Au electrodes, while the remaining two pins were connected to the Pt heater for temperature control. Gas‐sensing measurements were performed using a custom‐built test platform equipped with VIC‐K210 mass‐flow controllers and a Keysight DAQ970A data‐acquisition system, which recorded the resistance of each sensing channel once every 4 s. Prior to sensing, all sensors were stabilized for 3 h under baseline air at the target operating temperature. For concentration‐dependent sensing analysis, sensors were exposed to 100, 400, 700, and 1000 ppb of the three sulfur‐containing gases (H_2_S, CH_3_SH, (CH_3_)_2_S), along with five interfering gases (NO_2_, NH_3_, C_2_H_5_OH, CH_3_COCH_3_, and C_7_H_8_). The humidity was controlled by using a bubbler‐dilution humidity generator, while maintaining a constant total flow of 1000 sccm. Each sensing cycle consisted of 10 min exposure followed by 20 min purging with clean air.

## Author Contributions

S.E., D.K., and D.‐H.K. conceived the project. S.E., D.K., and D.‐H.K. wrote the manuscript. Synthesis, characterization, and chemical gas sensing measurements were carried out by S.E. under guidance of D.‐H. K. J.‐H.E. helped with gas sensing measurements. S.‐J.K., helped with characterization. M.S. helped with synthesis process. D.K. designed and implemented the deep‐ensemble Transformer architecture and evaluated the model performance. All authors discussed the results presented in this manuscript.

## Conflicts of Interest

The author declare no conflicts of interests.

## Supporting information




**Supporting File**: advs76134‐sup‐0001‐SuppMat.docx.

## Data Availability

The data that support the findings of this study are available from the corresponding author upon reasonable request.
