# Supplementary material for: Uncertainty‐Aware Deep Ensembles for Robust and Reliable Chemical Sensor Arrays
Source: Adv Sci (Weinh). 2026 Jun 15:e76134. Online ahead of print. doi: 10.1002/advs.76134 (PMC13336904; doi:10.1002/advs.76134)
Supplement: Supplementary file 1 — Supporting File: advs76134‐sup‐0001‐SuppMat.docx. [file ADVS-9999-e76134-s001.docx]

**Supplementary Information**

**Uncertainty-Aware Deep Ensembles for Robust and Reliable Chemical Sensor Arrays**

Sungwoo Eo^1^, Ji-Hwan Eum^1^, Suk-Jeong Kwon^1^, Minji Sagong^1^, Dongchan Kim^2,*^, and Dong-Ha Kim^1,*^

^1^Department of Materials Science and Chemical Engineering, Hanyang University, Ansan 15588, Republic of Korea

^2^Department of Artificial Intelligence, Hanyang University, Ansan 15588, Republic of Korea

^*^dongchan0507@hanyang.ac.kr, and dongha0507@hanyang.ac.kr

**Table of Contents**

**Supplementary Figure 1.** Experimental procedure for the synthesis of metal catalysts-decorated metal oxide nanofibers for gas sensor applications.

**Supplementary Figure 2.** SEM images of electrospun nanofibers before and after calcination.

**Supplementary Figure 3.** Real-time temperature profiles of (a) SnO_2_, (b) Co_3_O_4_, and (c) WO_3_ nanofibers deposited on glass substrates with different drop-coating counts during IPL irradiation.

**Supplementary Figure 4**. TEM and high-resolution TEM images of (a) pristine, (b) Pt-, (c) Pd-, (d) Ir-, and (e) Co-decorated WO_3_ nanofibers after IPL treatment.

**Supplementary Figure 5.** XRD patterns of (a) WO_3_, (b) SnO_2_, and (c) Co_3_O_4_ nanofibers before and after IPL treatment.

**Supplementary Figure 6.** XPS analysis of metal-catalyst-decorated WO­_3_ nanofibers in the vicinity of the W 4f region and the W^6+^/W^5+^ ratio.

**Supplementary Figure 7.** Resistance–time characteristics of the sensor array measured at various operating temperatures.

**Supplementary Figure 8.** Temperature-dependent H_2_S sensing characteristics of Pt@WO_3_ nanofibers.

**Supplementary** **Figure 9.** Dynamic H_2_S sensing responses of pristine and catalyst-decorated metal oxide nanofiber sensors under dry and humid conditions.

**Supplementary Figure 10.** Dynamic (CH_3_)_2_S sensing responses of pristine and catalyst-decorated metal oxide nanofiber sensors under dry and humid conditions.

**Supplementary Figure 11.** Dynamic CH_3_SH sensing responses of pristine and catalyst-decorated metal oxide nanofiber sensors under dry and humid conditions.

**Supplementary Figure 12.** Heatmaps showing the gas responses of various catalyst–decorated metal oxide nanofiber sensors toward different gases.

**Supplementary Figure 13.** Low-concentration VSC sensing response under humid conditions.

**Supplementary Figure 14.** Dynamic sensing responses toward binary VSC mixtures.

**Supplementary Figure 15.** Dynamic sensing responses toward ternary VSC mixture.

**Supplementary Figure 16.** Heatmaps showing the gas responses of various catalyst–metal-oxide nanofiber sensors toward single, binary, and ternary sulfur-compound conditions

**Supplementary Figure 17.** Response and recovery times of catalyst–decorated metal-oxide nanofiber sensors.

**Supplementary Figure 18.** Schematic illustration of the breath-matrix sensing setup using collected exhaled breath.

**Supplementary Figure 19.** Real-breath matrix validation of M@WO_3_ nanofiber sensors.

**Supplementary Figure 20.** Durability and humidity-dependent gas-sensing stability of the 15-channel catalyst-decorated metal oxide nanofiber sensor array toward three sulfur-containing gases.

**Supplementary Figure 21.** Sample-wise visualization of model outputs under two-gas mixture conditions.

**Supplementary Figure 22.** Sample-wise visualization of model outputs under three-gas mixture conditions.

**Supplementary Figure 23.** Uncertainty-aware regression results on the test set obtained from the deep-ensemble Transformer.

**Supplementary Figure 24.** Per-gas concentration prediction and classification results.

**Supplementary Figure 25.** Epistemic uncertainty analysis for uncertainty-aware classification on the test set.

**Supplementary Table 1.** Atomic compositions of catalyst-decorated WO_3_ nanofibers obtained from XPS analysis.

**Supplementary Table 2.** Ablation study on metal oxide sensing materials.

**Supplementary Table 3.** Ablation study on sliding-window size.

**Supplementary Table 4.** Per-gas classification performance comparison (precision/recall/F1).

**Supplementary Table 5.** Per-gas classification error count comparison (FP/FN).

**Supplementary Table 6.** Detailed electrospinning conditions for the synthesis of SnO_2_, Co_3_O_4_, and WO_3_ nanofibers.

**Supplementary Figure 1.** Experimental procedure for the fabrication of metal-catalyst-decorated metal oxide NFs-based sensor arrays for AI-integrated gas sensing applications. (a) Synthesis of metal oxide NFs via preparation of electrospinning precursor solution, electrospinning, and subsequent calcination in air. (b) IPL-driven photothermal activation for the decoration of catalytic nanoparticles on the surface of metal oxide NFs. (c) An AI-integrated gas-sensing framework integrating gas-sensing measurements with deep-learning-based gas-species classification and concentration quantification, alongside predictive uncertainty assessment.


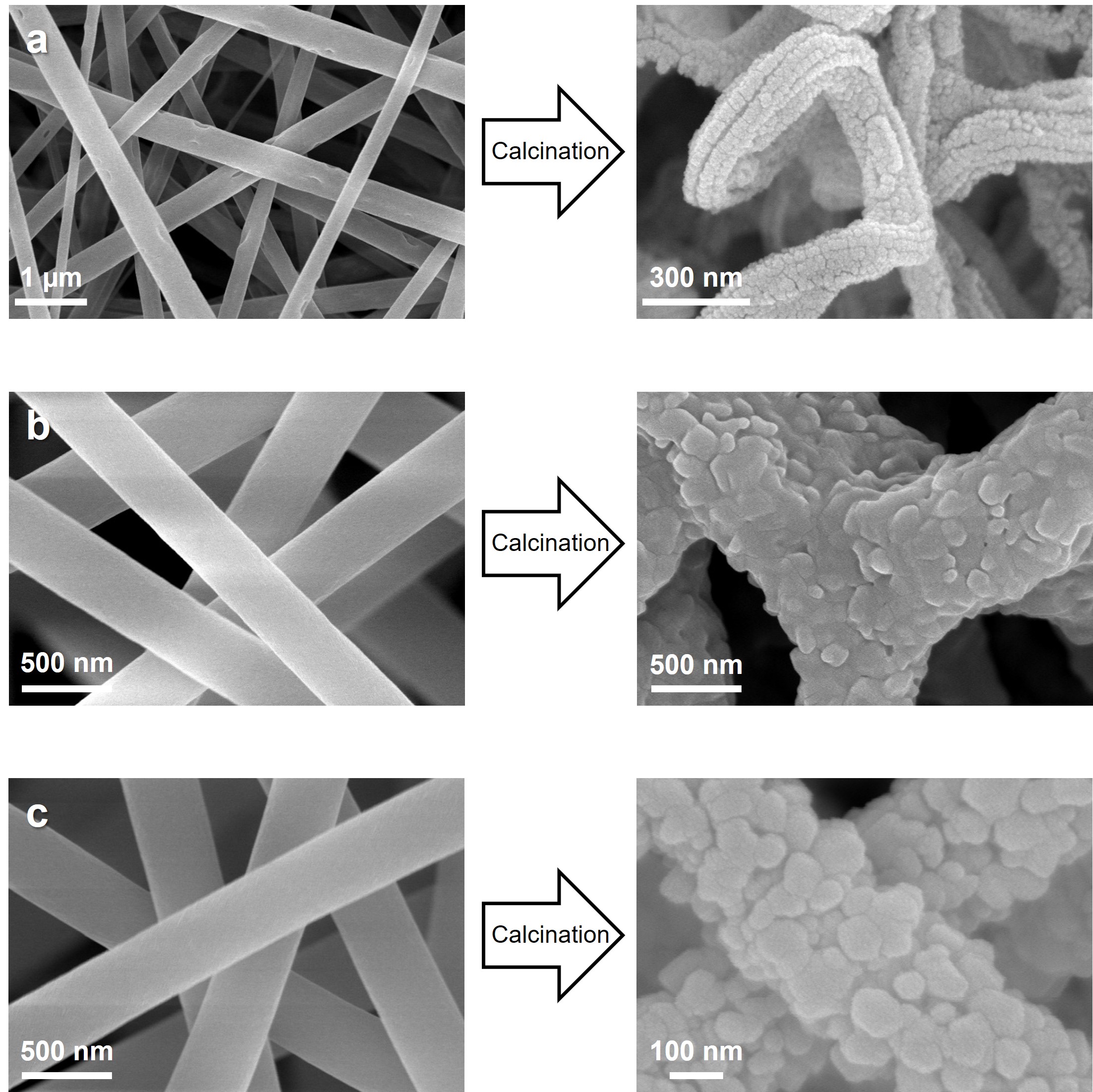


**Supplementary Figure 2.** SEM images of electrospun metal oxide NFs before and after calcination. (a) SnO_2_ NFs calcined at 500 °C for 1 h, (b) Co_3_O_4_ NFs calcined at 400 °C for 1 h, and (c) WO_3_ NFs calcined at 600 °C for 1 h. Each sample shows the morphological evolution from smooth polymeric NFs to crystalline oxide NFs with interconnected granular structures.


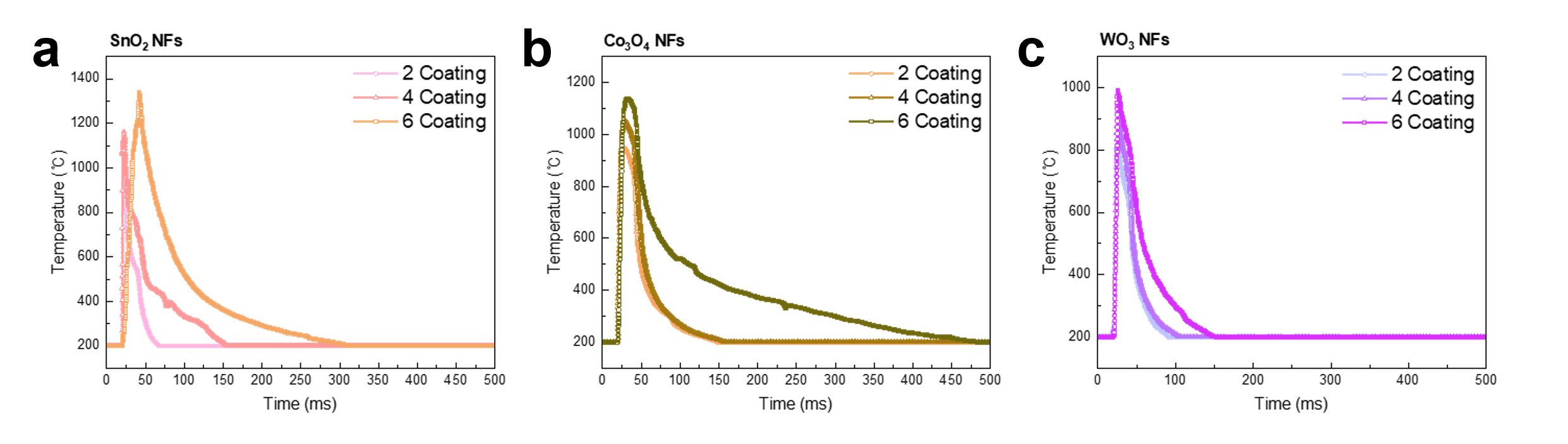


**Supplementary Figure 3.** Real-time temperature profiles during IPL irradiation. Temperature profiles of (a) SnO_2_ NFs, (b) Co_3_O_4_ NFs, and (c) WO_3_ NFs deposited on glass substrates with different drop-coating counts. The temperature rapidly increases to above 800–1300 °C within milliseconds and decays immediately after each light pulse. Increasing coating thickness results in higher peak temperatures; accordingly, 6 drop-coating cycles were selected as the optimized condition.

**
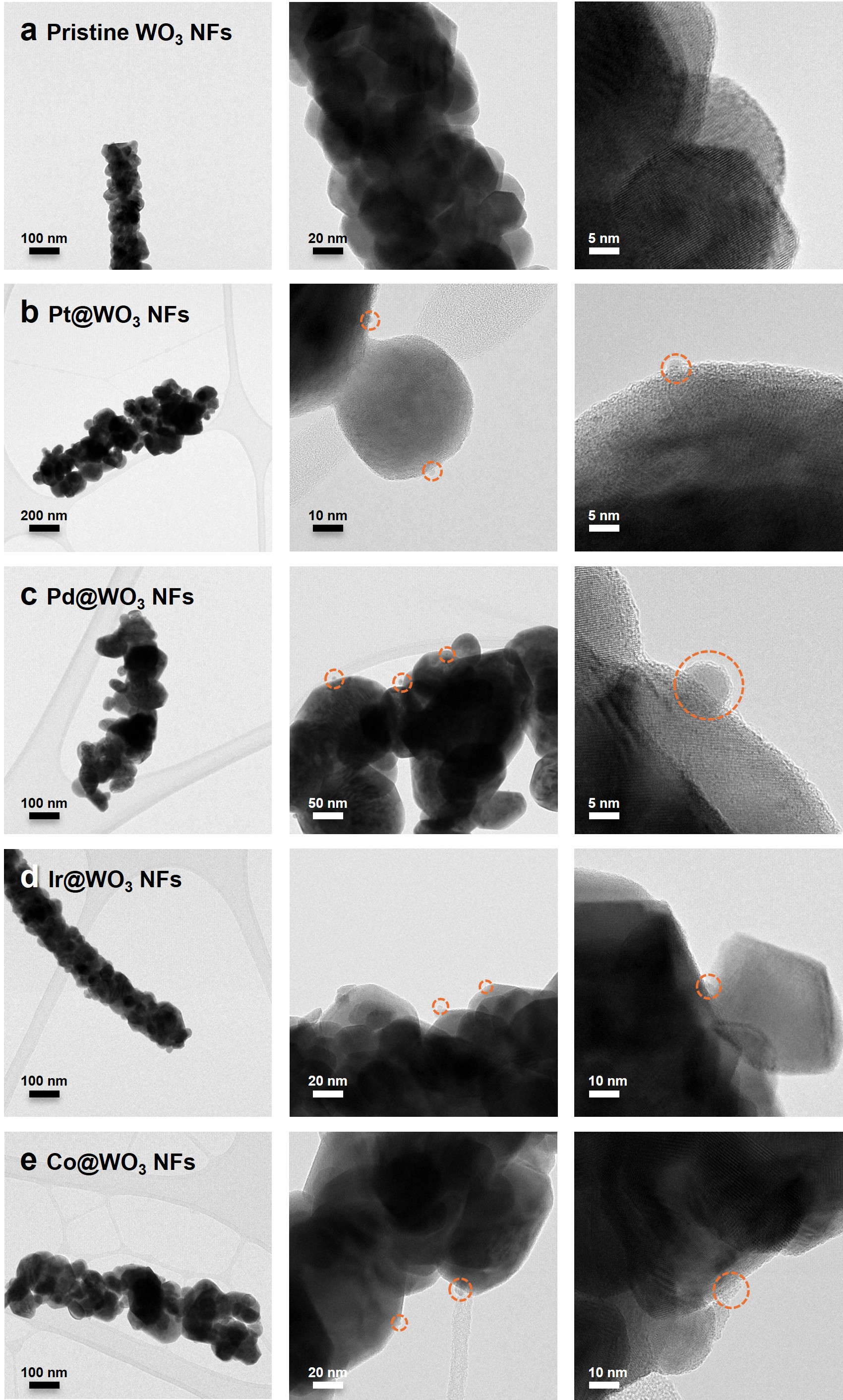
**

**Supplementary Figure 4**. TEM and HRTEM images. (a) Pristine WO­­_3_ NFs, (b) Pt@WO_3_ NFs, (c) Pd@WO_3_ NFs, (d) Ir@WO_3_ NFs, and (e) Co@WO_3_ NFs after IPL treatment. Metallic nanoparticles (highlighted by orange circles) are observed on the surface of the WO_3_ NFs.


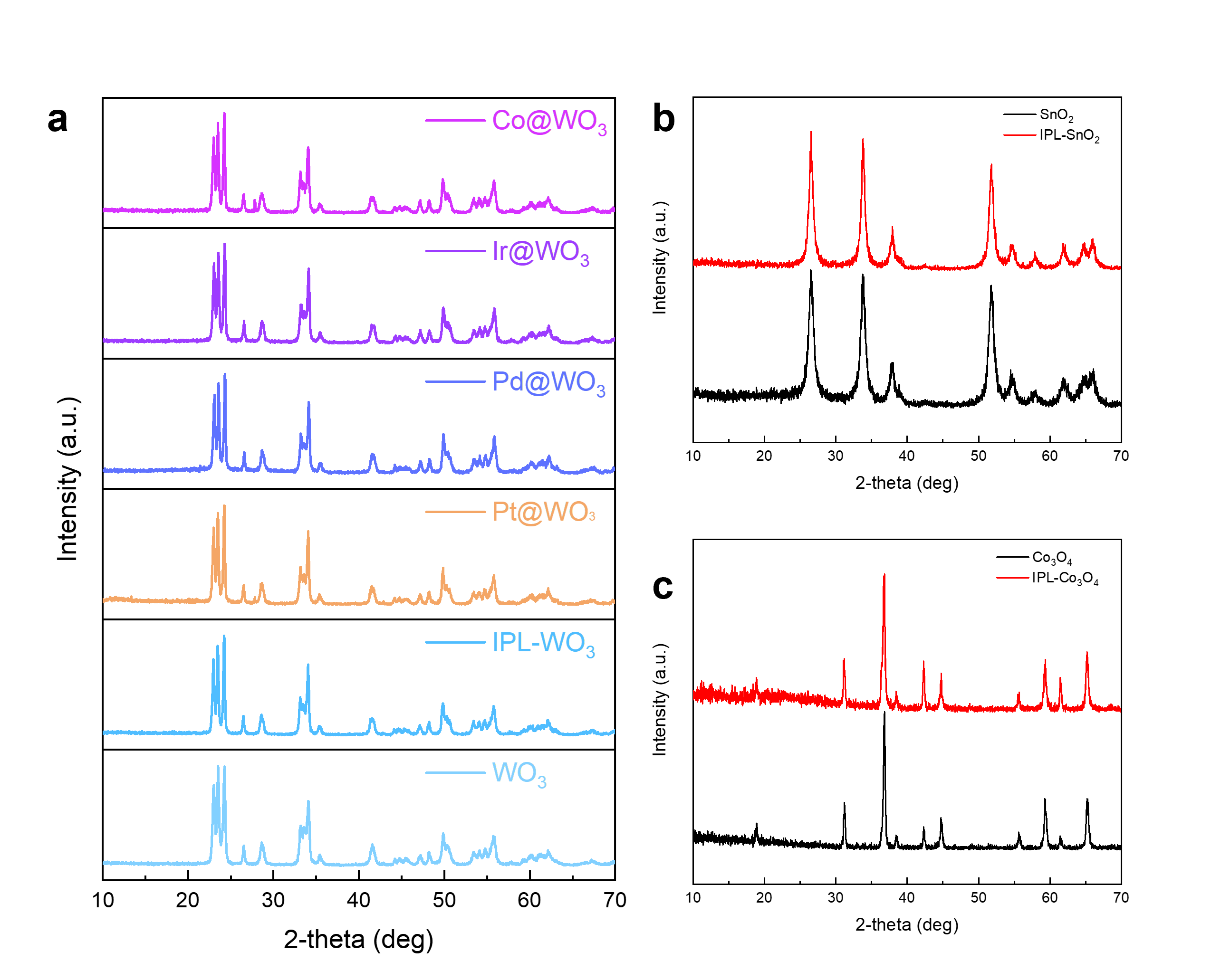
**Supplementary Figure 5.** XRD analysis of metal oxides NFs before and after IPL treatment. XRD patterns of (a) WO_3_ NFs decorated with different metal catalysts (none, Pt, Pd, Ir, and Co), (b) SnO_2_ NFs, and (c) Co_3_O_4_ NFs. High-temperature IPL treatment did not induce any distinct crystal phase evolution.

**Supplementary Figure 6.** XPS analysis of pristine and catalyst-decorated WO­_3_ NFs. (a) W 4f core-level XPS spectra of pristine WO­_3_ NFs and catalyst-decorated WO­_3_ NFs. (b) Corresponding area ratios of W^6+^/W^5+^ derived from the W 4f spectra.

**Supplementary Figure 7.** Resistance–time characteristics of the sensor array measured at different operating temperatures. Data obtained at (a) 200, (b) 240, (c) 290, and (d) 340 °C under exposure to 1 ppm H_2_S, NO_2_, NH_3_, and C_2_H_5_OH (EtOH).

**
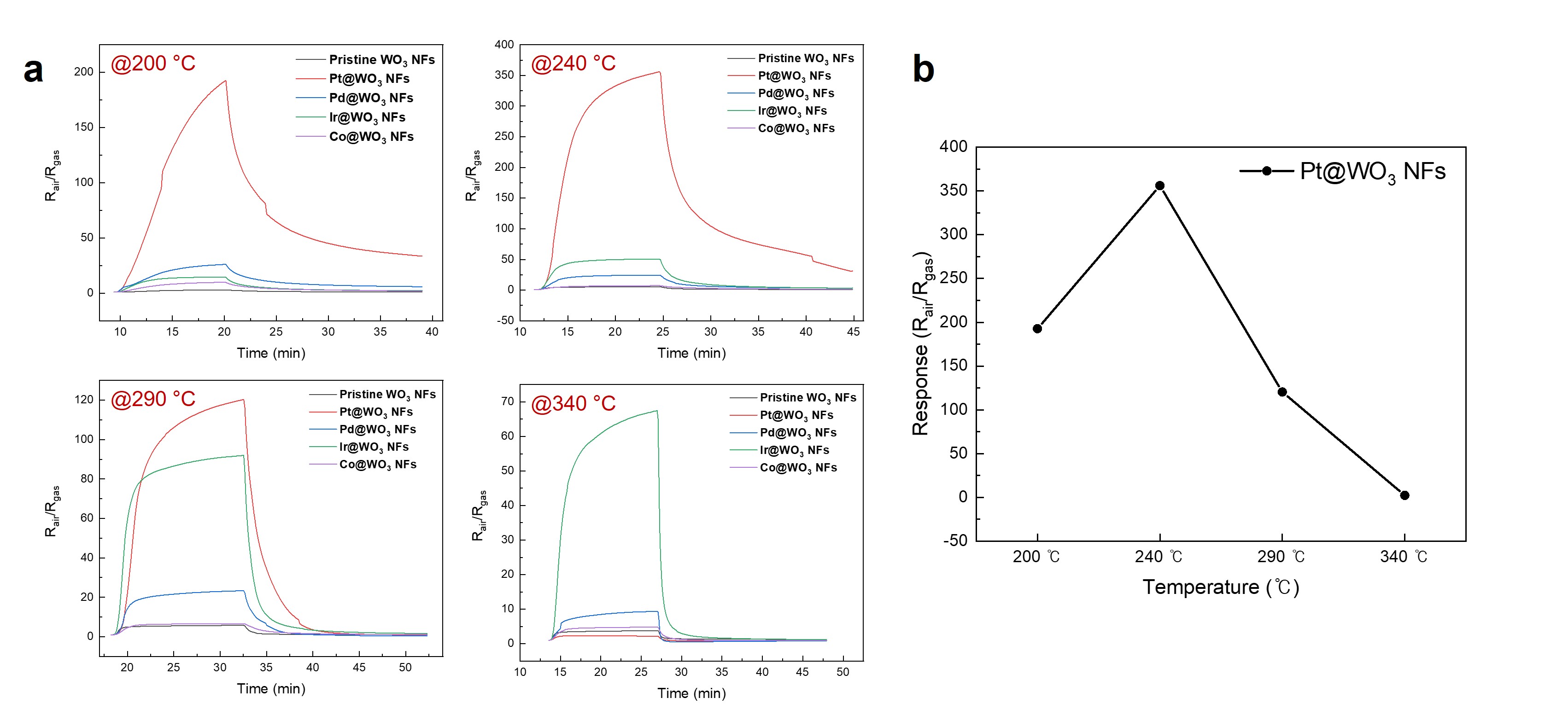
**

**Supplementary Figure 8.** Temperature-dependent H_2_S sensing characteristics. (a) H_2_S responses of pristine WO_3_ NFs and catalyst-decorated WO_3_ NFs measured at 200, 240, 290, and 340 °C, respectively. (b) H­_2_S responses of Pt@WO_3_ NFs at different operating temperatures, identifying 240 °C as the optimal operating temperature.

**Supplementary Figure 9.** Dynamic H_2_S gas sensing responses of pristine and catalyst-decorated metal oxide NFs under dry (0% RH) and humid (90% RH) conditions. H_2_S responses at (a) 0% RH and (b) 90% RH toward 100–1000 ppb.

**Supplementary Figure 10.** Dynamic (CH_3_)_2_S gas sensing responses of pristine and catalyst-decorated metal oxide NFs under dry (0% RH) and humid (90% RH) conditions. (CH_3_)_2_S responses at (a) 0% RH and (b) 90% RH toward 100–1000 ppb.

**Supplementary Figure 11.** Dynamic CH_3_SH gas sensing responses of pristine and catalyst-decorated metal oxide NFs under dry (0% RH) and humid (90% RH) conditions. CH_3_SH responses at (a) 0% RH and (b) 90% RH toward 100–1000 ppb.

**Supplementary Figure 12.** Heatmaps of normalized responses for catalyst-decorated metal oxide NFs sensors exposed to eight gas species, revealing the combined effects of gas identity, relative humidity (0% and 90% RH), and concentration (100 and 1000 ppb) on sensor performance.


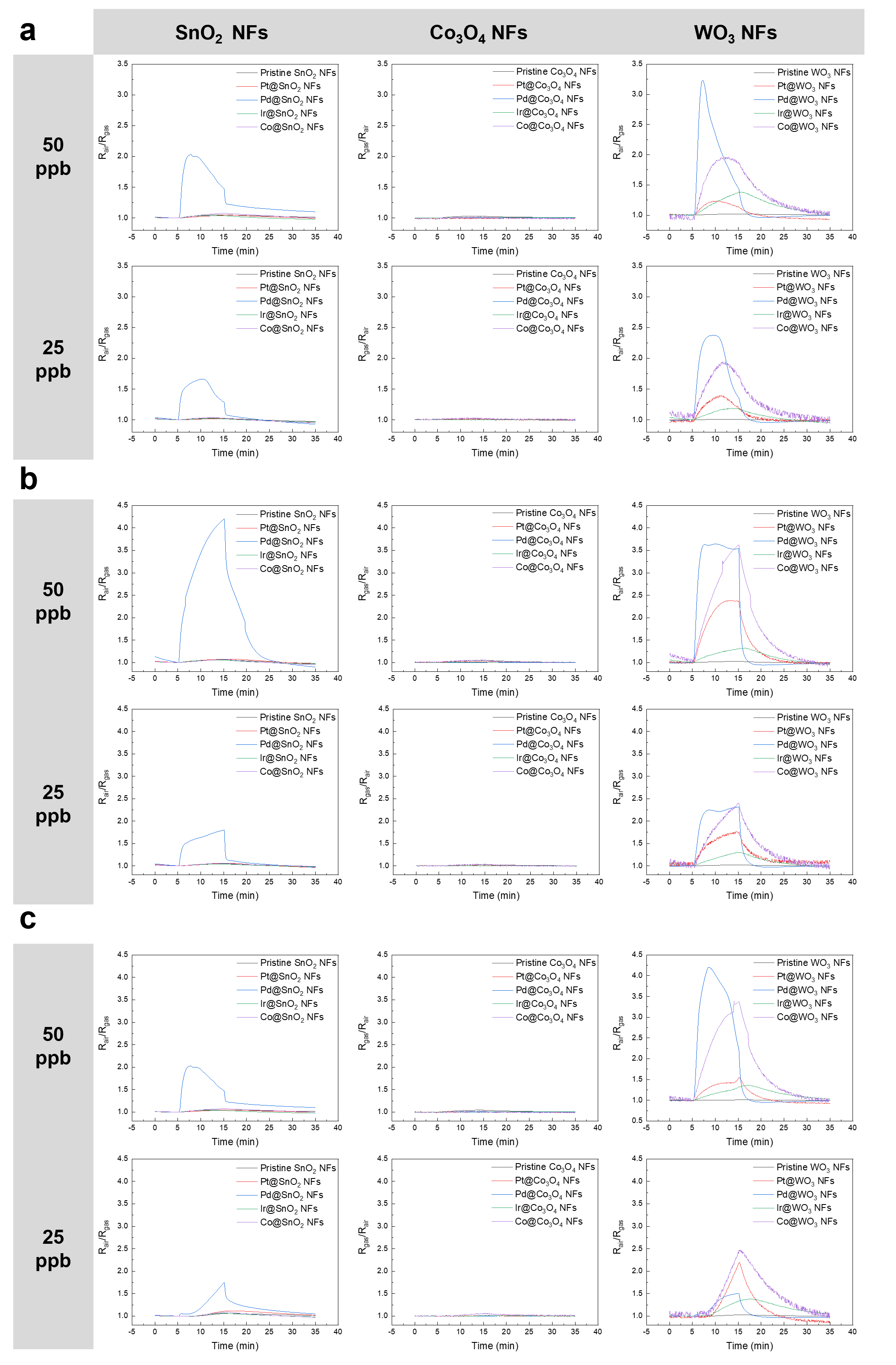


**Supplementary Figure 13.** Low-concentration VSC sensing responses under humid conditions. Dynamic sensing responses of the 15-channel catalyst-decorated metal oxide nanofiber sensor array toward (a) H_2_S, (b) (CH_3_)_2_S, and (c) CH_3_SH under 90% RH at 50 and 25 ppb. Each panel compares pristine, Pt-, Pd-, Ir-, and Co-decorated SnO_2_, Co_3_O_4_, and WO_3_ nanofiber sensors.


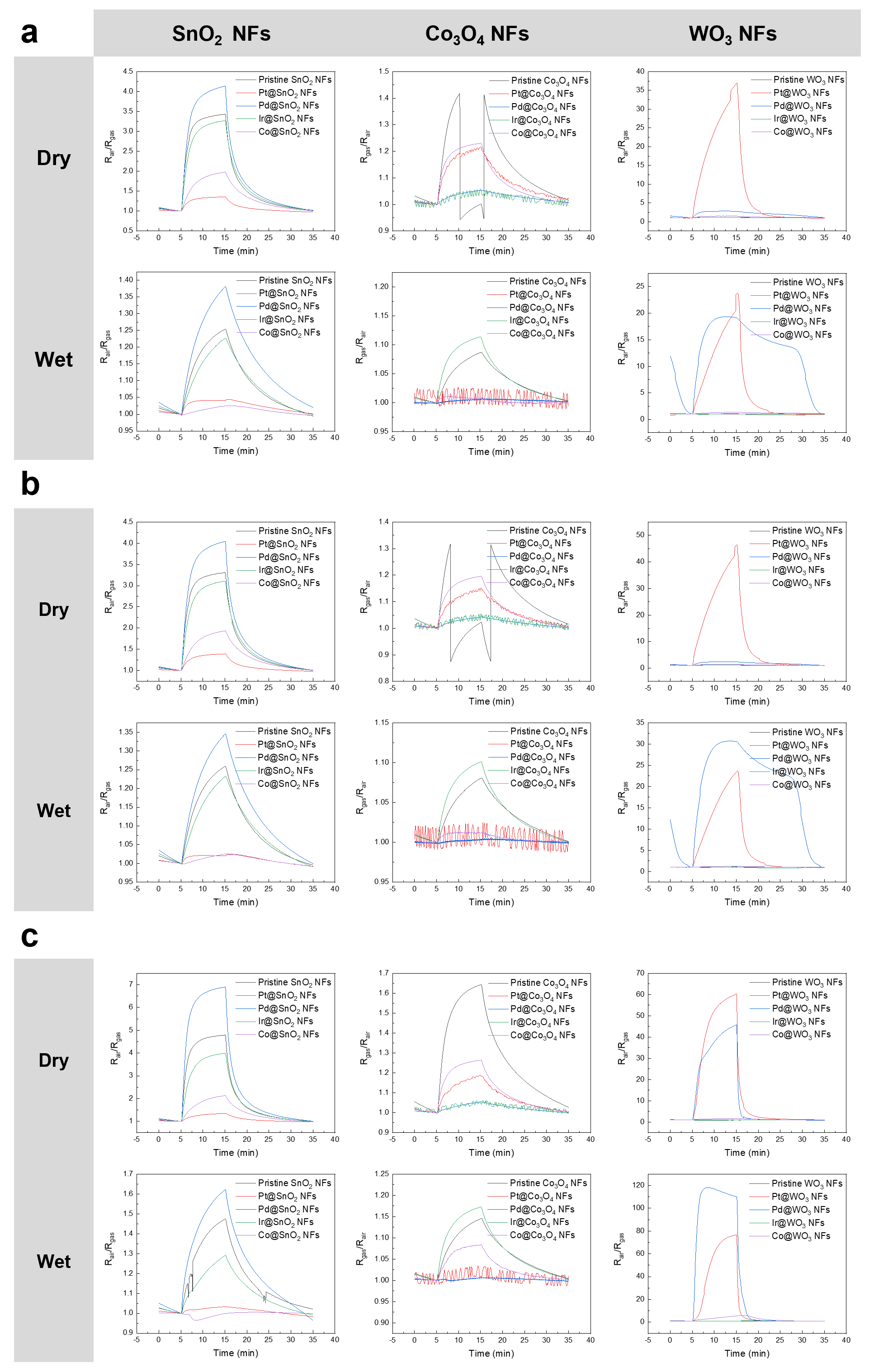


**Supplementary Figure 14.** Dynamic sensing responses toward binary VSC mixtures. Dynamic resistance responses of pristine and catalyst-decorated metal oxide nanofiber sensors to binary VSC mixtures under dry and humid conditions: (a) H_2_S + CH_3_SH, (b) (CH_3_)_2_S + CH_3_SH, and (c) H_2_S + (CH_3_)_2_S. Each gas was introduced at 1 ppm, giving a total VSC concentration of 2 ppm.


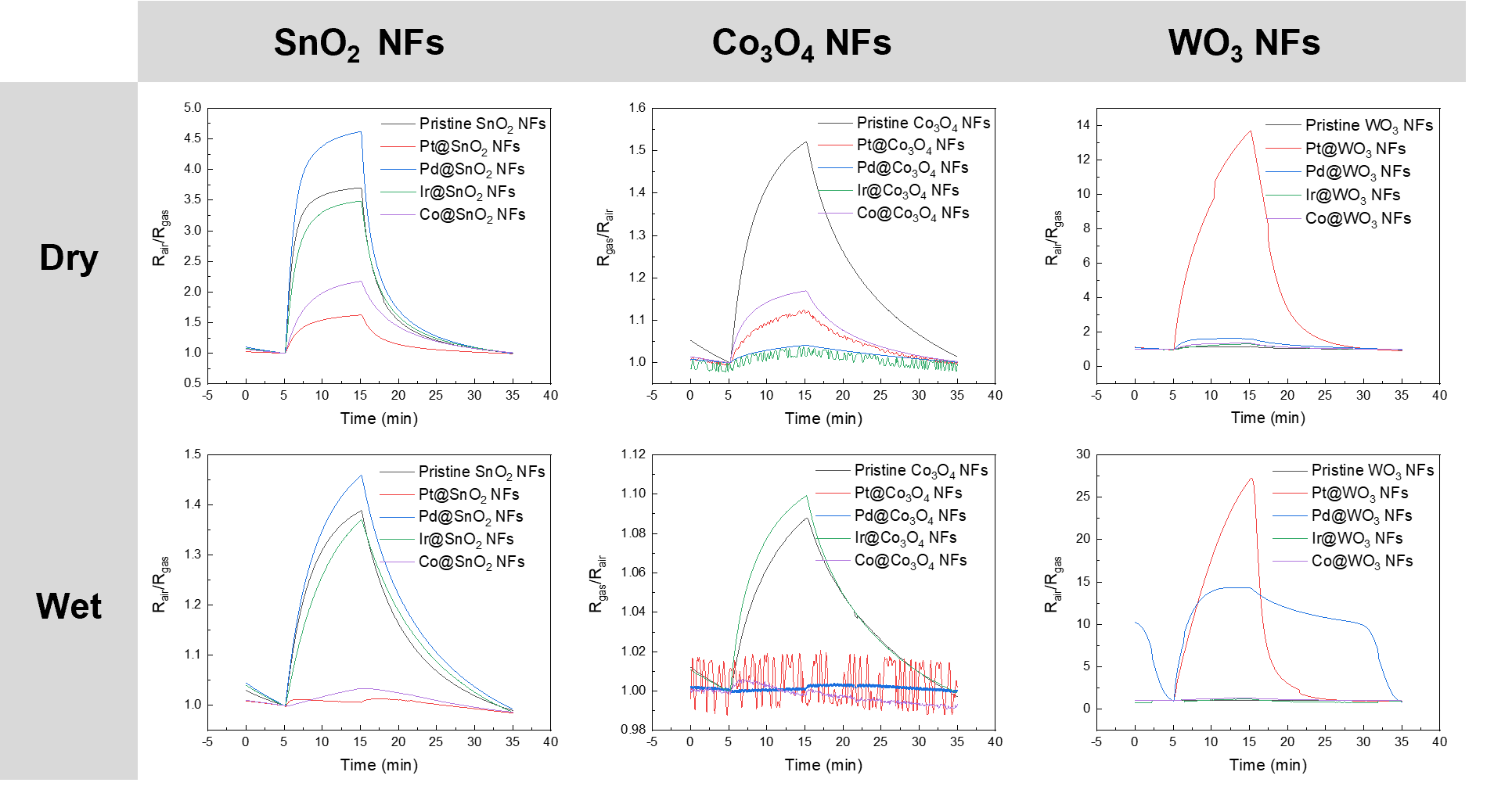


**Supplementary Figure 15.** Dynamic sensing responses toward ternary VSC mixture. Dynamic resistance responses of pristine and catalyst-decorated metal oxide nanofiber sensors to ternary VSC mixture under dry and humid conditions. H_2_S + CH_3_SH + (CH_3_)_2_S were each introduced at 1 ppm, giving a total VSC concentration of 3 ppm.

**
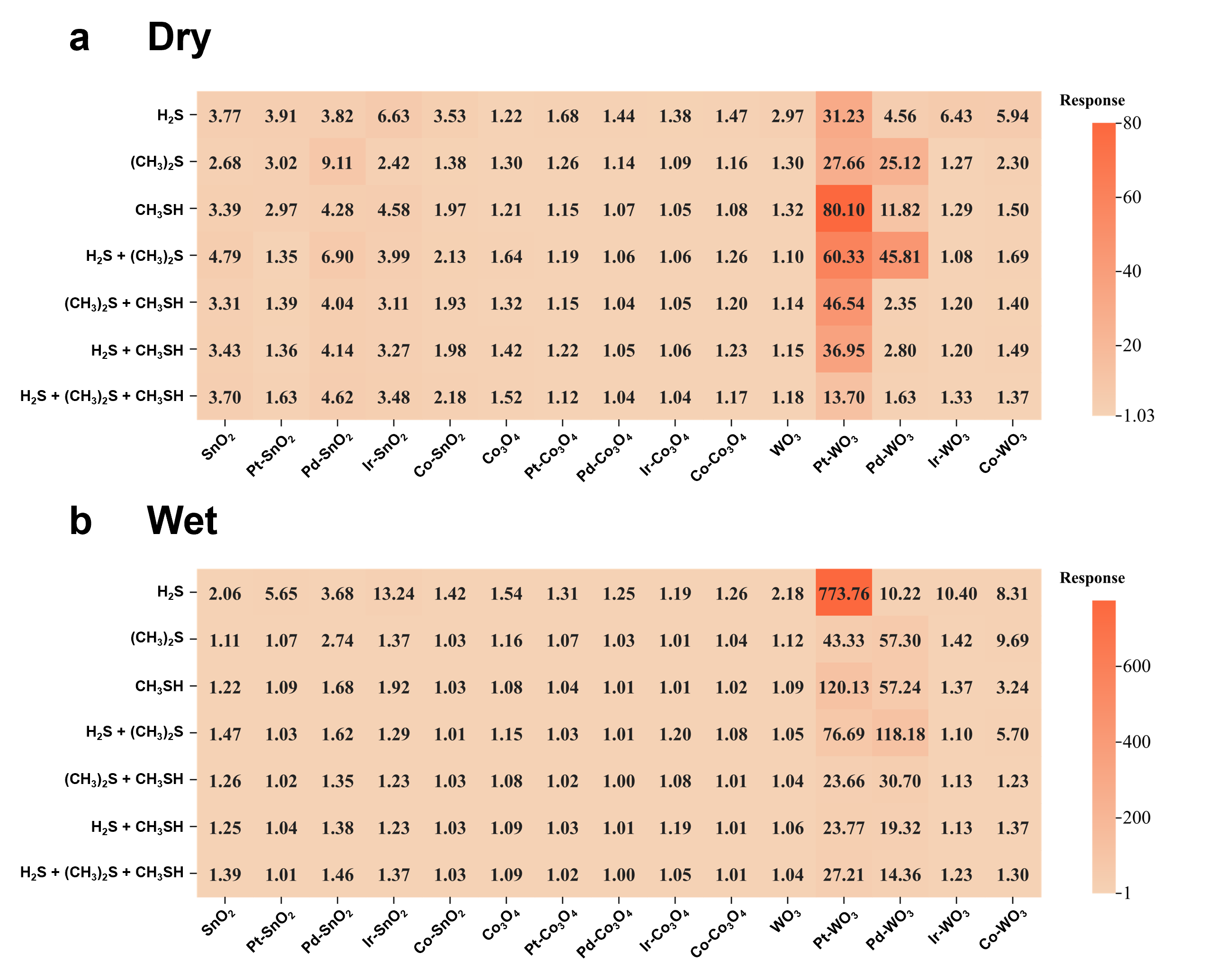
**

**Supplementary Figure 16.** Heatmaps showing the gas responses of various catalyst–metal-oxide nanofiber sensors toward single, binary, and ternary sulfur-compound conditions under (a) dry and (b) humid (wet) environments. Single-gas measurements were performed using individual sulfur compounds at 1 ppm, whereas binary and ternary mixtures were measured at total concentrations of 2 ppm and 3 ppm, respectively, corresponding to 1 ppm per component gas. Each cell represents the relative response ratio, highlighting composition- and humidity-dependent response patterns of the sensor array.


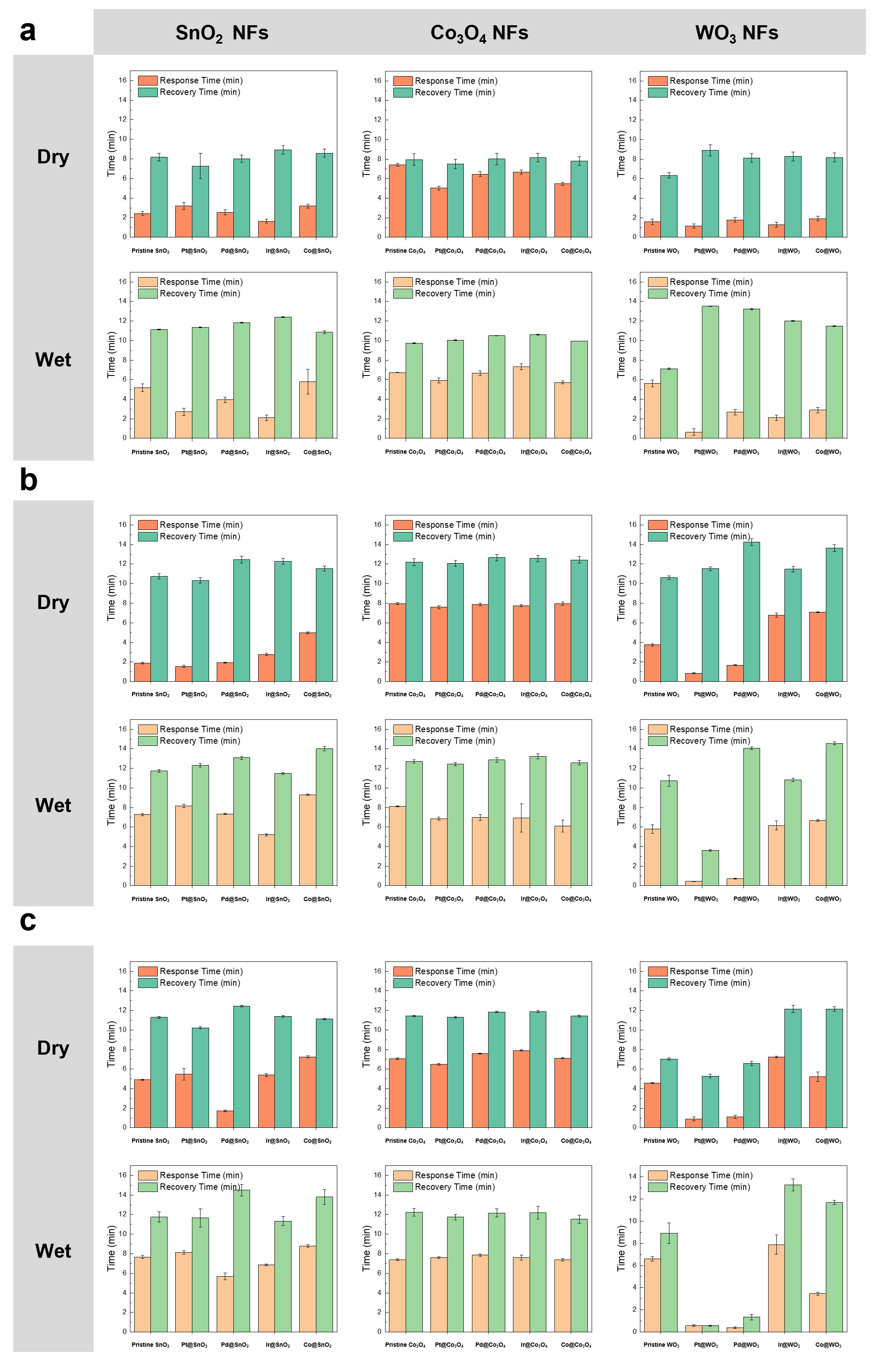


**Supplementary Figure 17.** Response and recovery times of catalyst–decorated metal-oxide nanofiber sensors toward (a) H_2_S, (b) CH_3_SH, and (c) (CH_3_)_2_S at 1 ppm under dry (0% RH) and humid (90% RH) conditions. Orange and green bars represent response and recovery times, respectively. Values are presented as mean ± standard deviation (n = 5). The observed gas-dependent kinetic differences indicate rapid sensing behavior and support the feasibility of fast gas identification despite the longer data acquisition window used in this study.


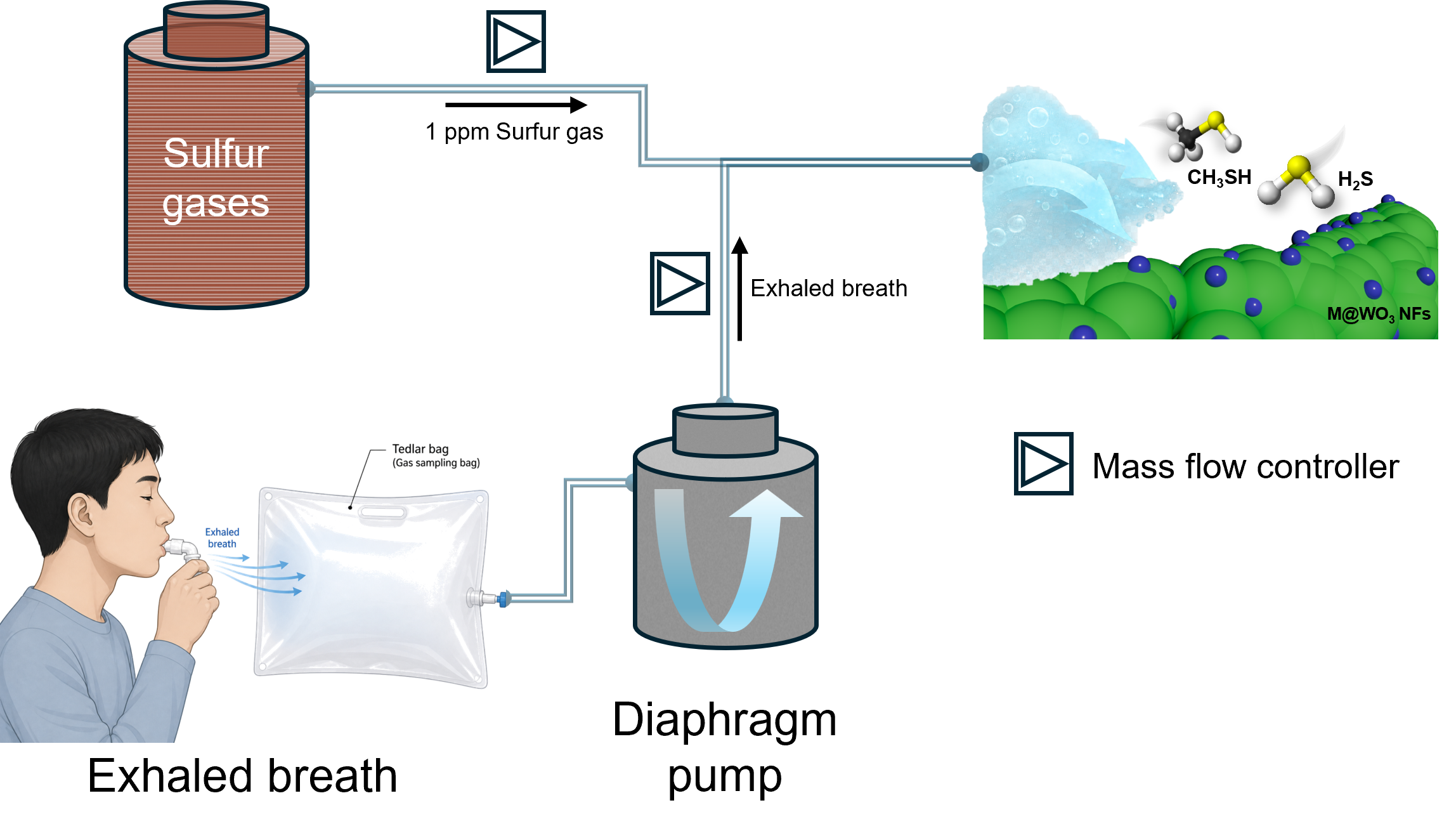


**Supplementary Figure 18.** Schematic illustration of the breath-matrix sensing setup using collected exhaled breath. Exhaled breath was collected in a Tedlar bag and delivered using a diaphragm pump. The exhaled-breath stream was mixed with sulfur-containing gases to generate breath-matrix test conditions before exposure to the M@WO_3_ NF-based sensor.


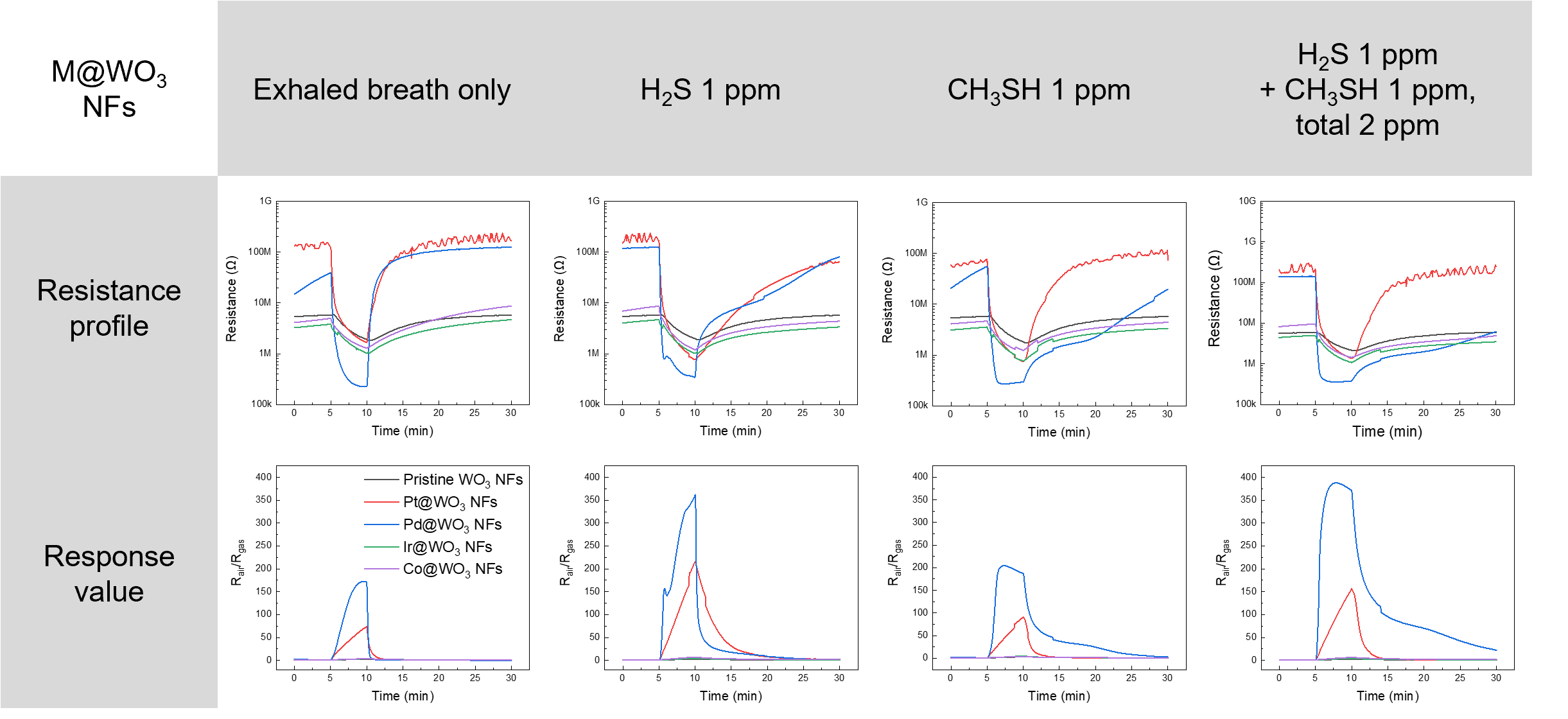


**Supplementary Figure 19.** Real-breath matrix validation of M@WO_3_ nanofiber sensors. Resistance profiles and corresponding response values of pristine and catalyst-decorated WO_3_ NF sensors measured under exhaled-breath-derived conditions. The test conditions included exhaled breath only, exhaled breath with 1 ppm H_2_S, exhaled breath with 1 ppm CH_3_SH, and exhaled breath with a binary mixture of 1 ppm H_2_S and 1 ppm CH_3_SH, corresponding to a total VSC concentration of 2 ppm. These results demonstrate that the M@WO_3_ NF sensors retain distinguishable VSC-responsive behavior under a breath-derived matrix.

**Supplementary Figure 20.** Long-term cyclic stability of the 15-channel catalyst-decorated metal oxide NFs sensor array over 20 exposure-recovery cycles (1 ppm sulfur-containing gases) for (a) H_2_S, (b) (CH_3_)_2_S, and (c) CH_3_SH under dry (0% RH) and humid (90% RH) environments.


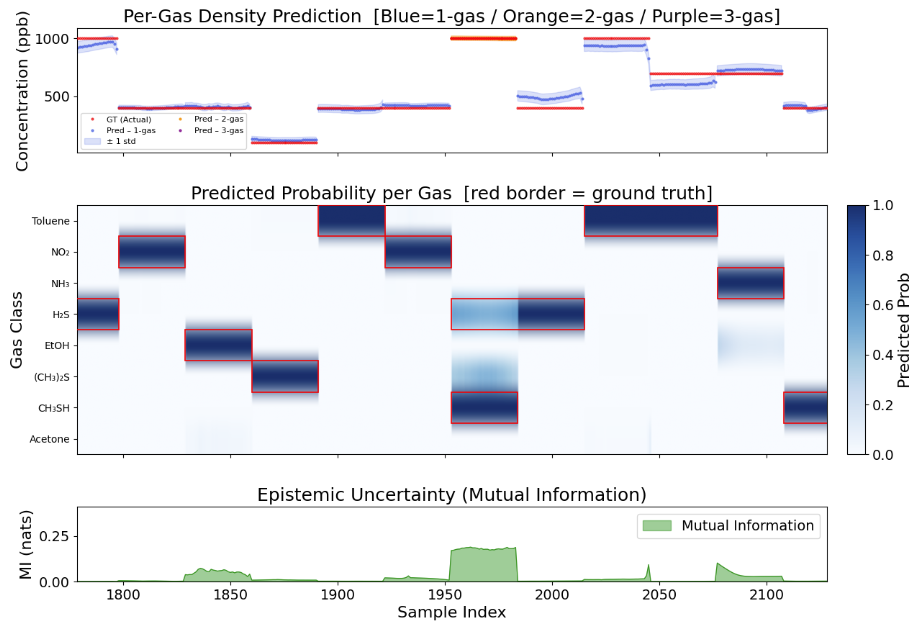


**Supplementary Figure 21.** Sample-wise visualization of model outputs under two-gas mixture conditions. The top panel compares the ground-truth and predicted gas concentrations across sample indices, with blue, orange, and purple markers denoting single-gas, two-gas mixture, and three-gas mixture samples, respectively. The shaded area indicates one standard deviation of the predicted concentration. The middle panel shows the predicted probability for each gas class, with red boxes indicating ground-truth labels. Since mixture samples contain multiple gases, one sample index may correspond to more than one ground-truth label. The bottom panel presents the epistemic uncertainty quantified by mutual information (MI). Regions with more dispersed predicted probabilities, such as around sample indices 1970 and 2100, show increased MI values, indicating higher epistemic uncertainty. The figure provides a sample-wise view of concentration estimation, multi-label gas identification, and uncertainty estimation under single-gas and mixture conditions.


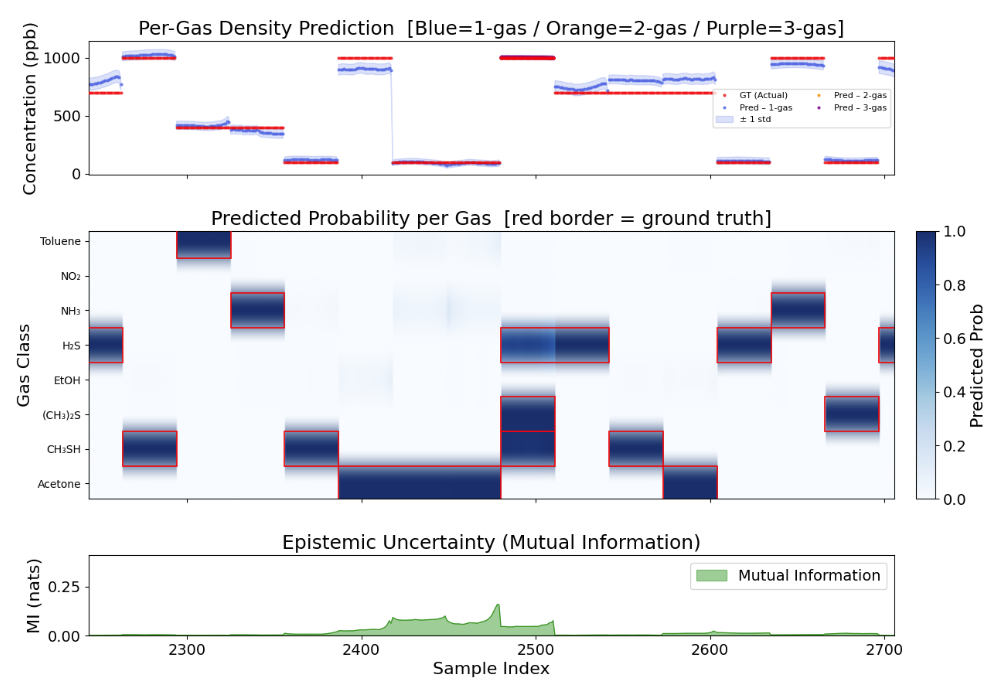


**Supplementary Figure 22.** Sample-wise visualization of model outputs under three-gas mixture conditions. This figure provides an additional example of the joint visualization shown in Supplementary Figure 17, focusing on samples that include three target gases. In the middle panel, multiple red boxes at the same sample indices indicate the simultaneous presence of three ground-truth gas labels. The predicted probability map shows that the model assigns high probabilities to the corresponding gas classes while suppressing non-target gases in most regions. The bottom panel shows that mutual information (MI) remains generally low, with localized increases observed near regions where the predicted probabilities become more dispersed across gas classes. This example further illustrates the model outputs for concentration estimation, multi-label gas identification, and epistemic uncertainty under three-gas mixture conditions.


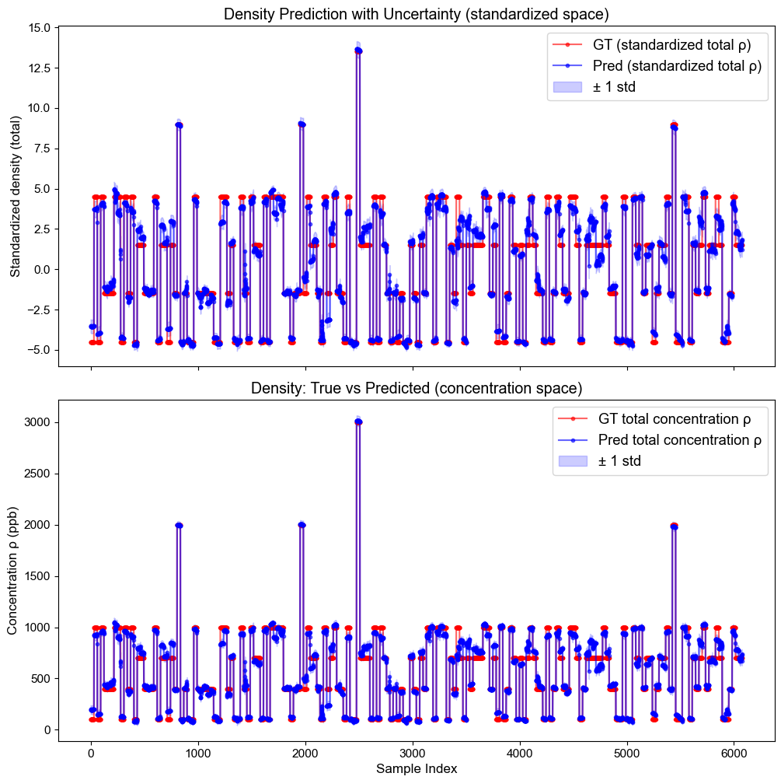


**Supplementary Figure 23.** Uncertainty-aware total concentration regression results on the test set.
Regression results for total gas concentration prediction obtained from the deep-ensemble Transformer. For multi-gas samples, the total concentration $\rho_{total}$ is computed by summing the concentrations of all present gas components; therefore, binary and ternary gas-mixture samples can have total concentrations of 2000 ppb and 3000 ppb, respectively. The top panel shows the ground-truth and predicted total concentrations in standardized space, while the bottom panel reports the corresponding predictions transformed back to the original concentration space ($\rho_{total}$, ppb). In both panels, the solid lines indicate the ensemble-mean prediction, and the shaded regions denote the ±1 standard deviation derived from the ensemble predictive variance, reflecting combined aleatoric and epistemic uncertainty.


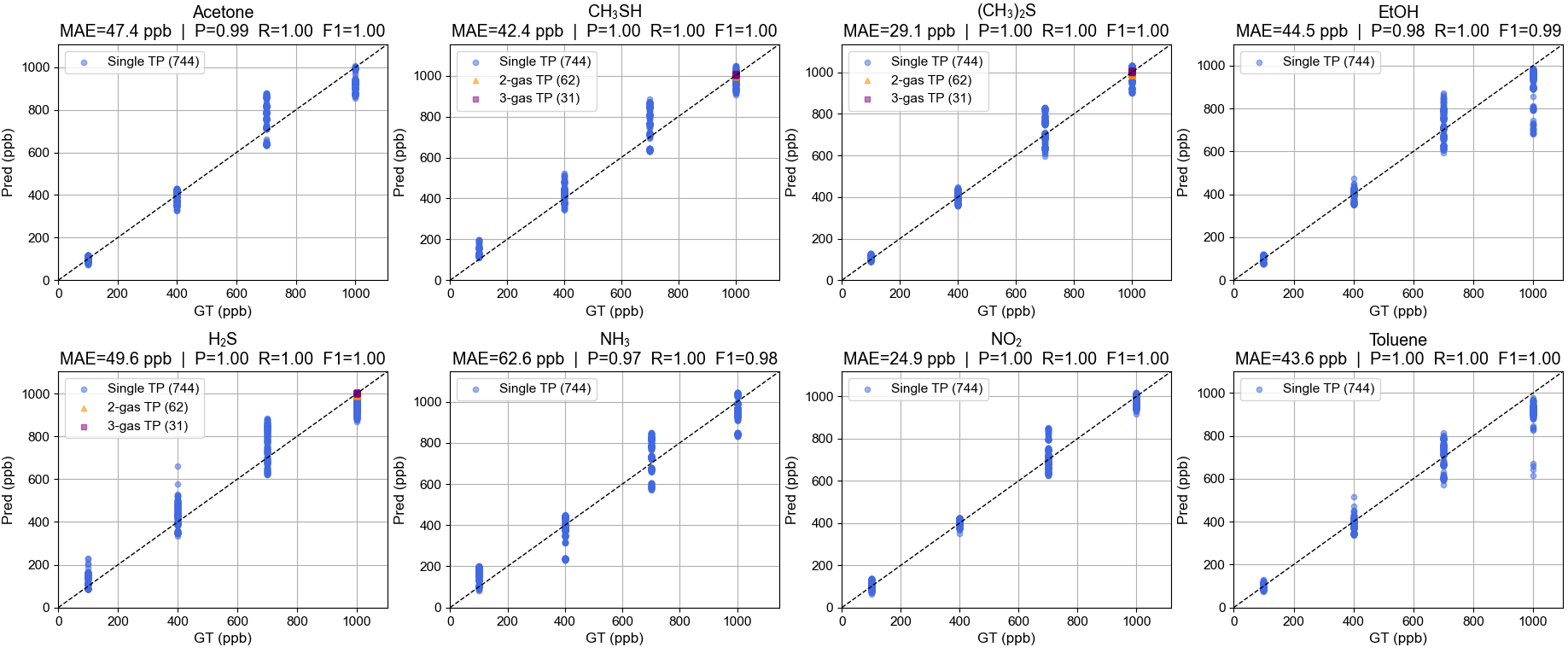


**Supplementary Figure 24.** Per-gas concentration prediction and classification results. Predicted concentrations are plotted against ground-truth concentrations for eight target gases. The dashed diagonal line represents the ideal prediction. Blue circles, orange upward triangles, and purple rectangles indicate true-positive detections in single-gas, two-gas mixture, and three-gas mixture samples, respectively. Each panel reports the mean absolute error (MAE) for concentration regression and the precision (P), recall (R), and F1-score for gas classification. The figure summarizes the per-gas performance of the model for concentration estimation and gas identification under single-gas and mixture conditions.


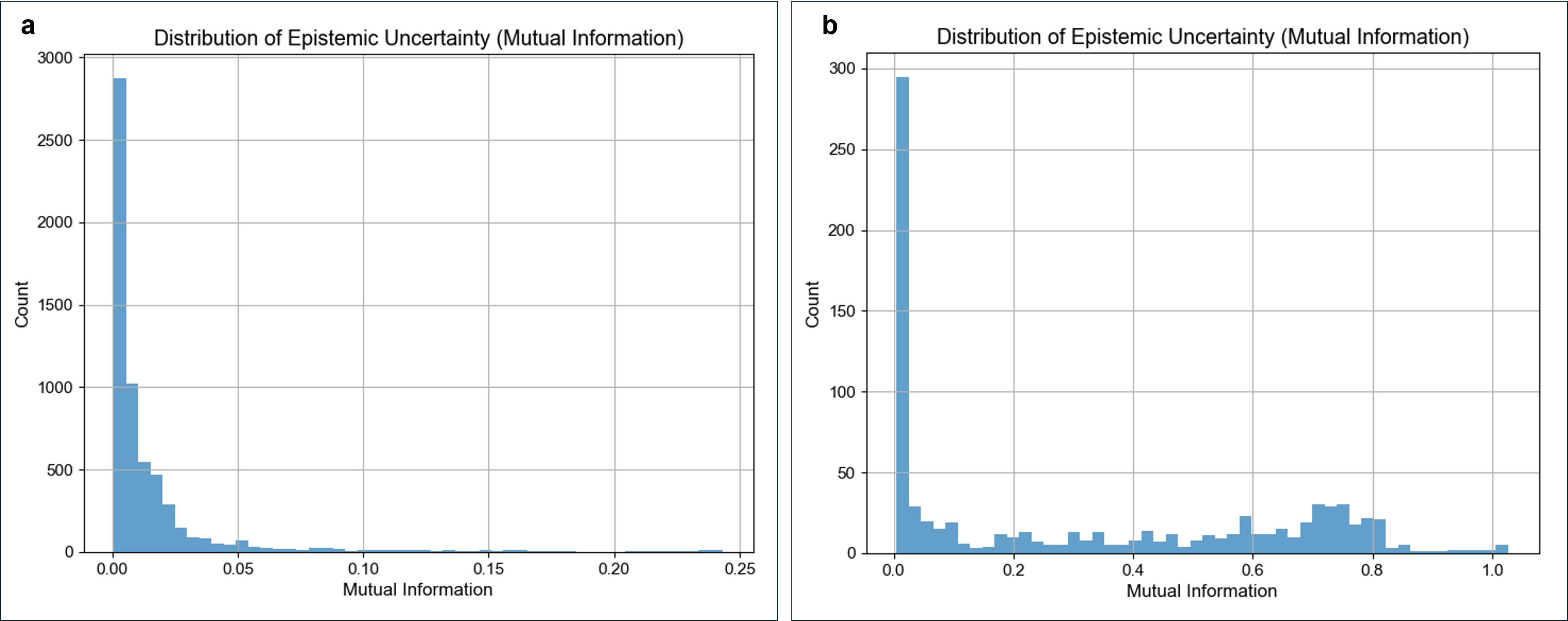


**Supplementary Figure 25.** Epistemic uncertainty analysis for uncertainty-aware classification on the test set. Distribution of epistemic uncertainty quantified by the mutual information (MI) between the predictive class distribution and ensemble members, obtained from the deep-ensemble Transformer. (a) In-distribution (ID) test set, where all eight gas classes are included during training. Most samples exhibit low MI values, indicating strong agreement among ensemble members and high model confidence. (b) Out-of-distribution (OOD) evaluation, where the model is trained on seven gas classes and tested on the unseen class (H_2_S). The MI distribution shifts toward substantially higher values, reflecting increased epistemic uncertainty due to disagreement among ensemble predictions when encountering an unknown gas. Higher MI indicates reduced model confidence and effective detection of OOD inputs.

**`**


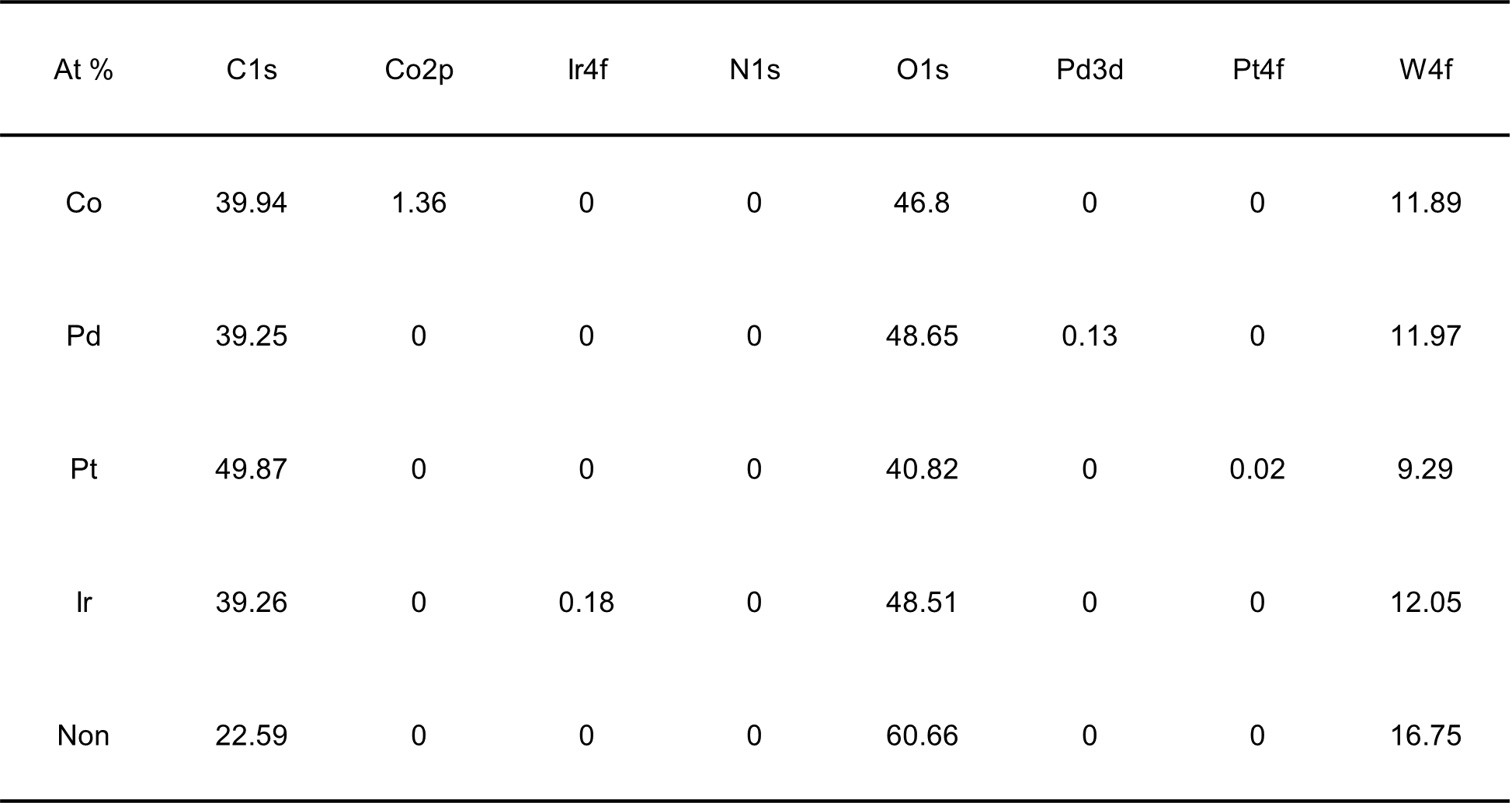
**Supplementary Table 1.** Atomic compositions of catalyst-decorated WO_3_ NFs obtained from XPS analysis.

**Supplementary Table 2.** Ablation study on metal oxide sensing materials.

| Metal oxide composition | Regression | | Classification | | | |
| --- | --- | --- | --- | --- | --- | --- |
|  | MAE (ppm) | RMSE (ppm) | Acc (%) | F1-micro (%) | $\mathrm{NLL}_{\mathrm{cls}}$ (-) | Brier (-) |
| SnO_2_ | 0.070 | 0.093 | 83.92 | 92.02 | 0.0514 | 0.0149 |
| SnO_2_ + Co_3_O_4_ | 0.050 | 0.066 | 96.15 | 99.45 | 0.0083 | 0.0014 |
| SnO_2_ + Co_3_O_4_ + WO_3_ (Proposed) | **0.045** | **0.062** | **99.23** | **99.62** | **0.0057** | **0.0010** |

**Supplementary Table 3.** Ablation study on sliding-window size.

| Sliding-window (min) | Regression | | Classification | | | |
| --- | --- | --- | --- | --- | --- | --- |
|  | MAE (ppm) | RMSE (ppm) | Acc (%) | F1-micro (%) | $\mathrm{NLL}_{\mathrm{cls}}$ (-) | Brier (-) |
| 5 | 0.065 | 0.084 | 99.15 | 99.58 | 0.0048 | **0.0009** |
| 10 | 0.056 | 0.076 | 99.10 | 99.54 | **0.0047** | **0.0009** |
| 20 (proposed) | **0.045** | **0.062** | **99.23** | **99.62** | 0.0057 | 0.0010 |
| 25 | 0.056 | 0.071 | 97.40 | 98.75 | 0.0104 | 0.0019 |

**Supplementary Table 4.** Per-gas classification performance comparison (precision/recall/F1).

| **Gas** | **Support** | **MLP** | | | **LSTM** | | | **Single Transformer** | | | **Proposed** | | |
| --- | --- | --- | --- | --- | --- | --- | --- | --- | --- | --- | --- | --- | --- |
|  |  | **P** | **R** | **F1** | **P** | **R** | **F1** | **P** | **R** | **F1** | **P** | **R** | **F1** |
| Acetone | 744 | 0.725 | 1.000 | 0.841 | 0.983 | 1.000 | 0.991 | 0.995 | 1.000 | 0.997 | 0.992 | 1.000 | 0.996 |
| CH₃SH | 837 | 1.000 | 1.000 | 1.000 | 1.000 | 1.000 | 1.000 | 1.000 | 1.000 | 1.000 | 1.000 | 1.000 | 1.000 |
| (CH₃)₂S | 837 | 1.000 | 0.989 | 0.995 | 0.964 | 1.000 | 0.982 | 0.964 | 1.000 | 0.982 | 1.000 | 1.000 | 1.000 |
| EtOH | 744 | 0.751 | 1.000 | 0.858 | 0.856 | 1.000 | 0.923 | 0.979 | 1.000 | 0.989 | 0.982 | 1.000 | 0.991 |
| H₂S | 837 | 0.964 | 1.000 | 0.982 | 0.964 | 1.000 | 0.982 | 0.963 | 0.963 | 0.963 | 0.999 | 1.000 | 0.999 |
| NH₃ | 744 | 0.768 | 1.000 | 0.869 | 0.973 | 1.000 | 0.986 | 0.983 | 1.000 | 0.991 | 0.966 | 1.000 | 0.983 |
| NO₂ | 744 | 0.885 | 1.000 | 0.939 | 0.983 | 1.000 | 0.991 | 1.000 | 1.000 | 1.000 | 1.000 | 1.000 | 1.000 |
| Toluene | 744 | 1.000 | 1.000 | 1.000 | 1.000 | 1.000 | 1.000 | 1.000 | 1.000 | 1.000 | 1.000 | 1.000 | 1.000 |
| **Macro avg.** | **-** | **0.887** | **0.999** | **0.935** | **0.965** | **1.000** | **0.982** | **0.986** | **0.995** | **0.990** | **0.992** | **1.000** | **0.996** |

**Supplementary Table 5.** Per-gas classification error count comparison (FP/FN).

| **Gas** | **MLP** | | **LSTM** | | **Single Transformer** | | **Proposed** | |
| --- | --- | --- | --- | --- | --- | --- | --- | --- |
|  | **FP** | **FN** | **FP** | **FN** | **FP** | **FN** | **FP** | **FN** |
| Acetone | 282 | 0 | 13 | 0 | 4 | 0 | 6 | 0 |
| CH₃SH | 0 | 0 | 0 | 0 | 0 | 0 | 0 | 0 |
| (CH₃)₂S | 0 | 9 | 31 | 0 | 31 | 0 | 0 | 0 |
| EtOH | 247 | 0 | 125 | 0 | 16 | 0 | 14 | 0 |
| H₂S | 31 | 0 | 31 | 0 | 31 | 31 | 1 | 0 |
| NH₃ | 225 | 0 | 21 | 0 | 13 | 0 | 26 | 0 |
| NO₂ | 97 | 0 | 13 | 0 | 0 | 0 | 0 | 0 |
| Toluene | 0 | 0 | 0 | 0 | 0 | 0 | 0 | 0 |
| **Total** | **882** | **9** | **234** | **0** | **95** | **31** | **47** | **0** |

**Supplementary Table 6.** Composition and electrospinning conditions for each metal oxide NFs.

**
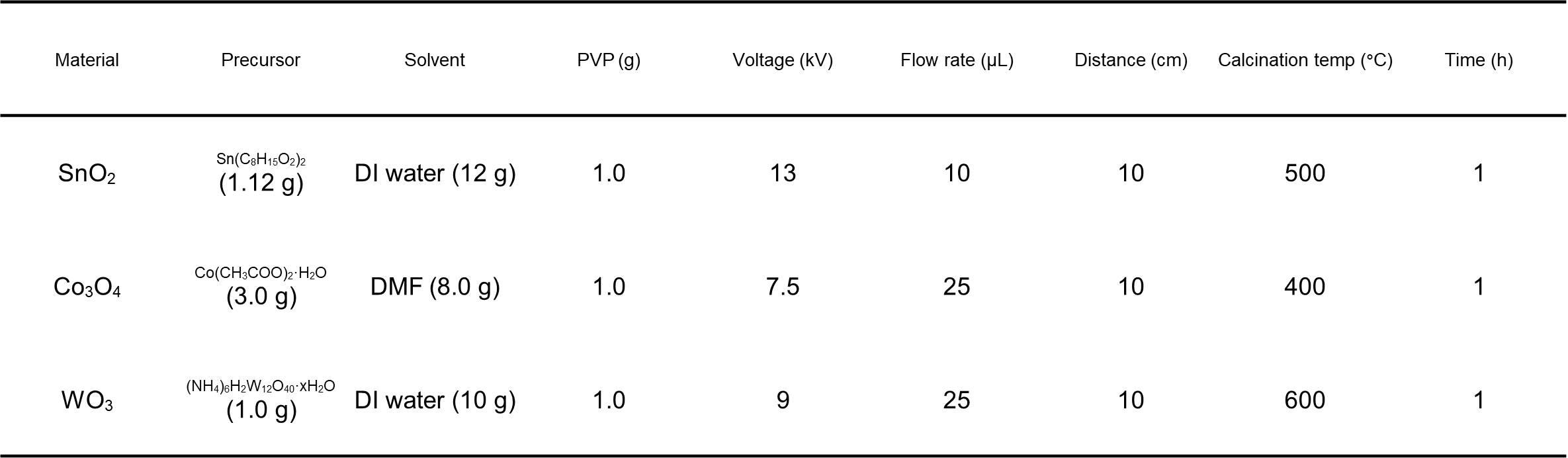
**
